# Targeting the LOX/hypoxia axis reverses many of the features that make pancreatic cancer deadly: inhibition of LOX abrogates metastasis and enhances drug efficacy

**DOI:** 10.15252/emmm.201404827

**Published:** 2015-06-15

**Authors:** Bryan W Miller, Jennifer P Morton, Mark Pinese, Grazia Saturno, Nigel B Jamieson, Ewan McGhee, Paul Timpson, Joshua Leach, Lynn McGarry, Emma Shanks, Peter Bailey, David Chang, Karin Oien, Saadia Karim, Amy Au, Colin Steele, Christopher Ross Carter, Colin McKay, Kurt Anderson, Thomas R Jeffry Evans, Richard Marais, Caroline Springer, Andrew Biankin, Janine T Erler, Owen J Sansom

**Affiliations:** 1 Cancer Research UK Beatson Institute, Garscube EstateGlasgow, UK; 2 The Garvan Institute of Medical ResearchSydney, NSW, Australia; 3 Cancer Research UK Manchester InstituteWithington, Manchester, UK; 4 West of Scotland Pancreatic Unit, Glasgow Royal InfirmaryGlasgow, UK; 5 Institute of Cancer Sciences, University of Glasgow, Garscube EstateGlasgow, UK; 6 Institute of Cancer ResearchLondon, UK; 7 Biotech Research & Innovation Centre (BRIC), University of CopenhagenCopenhagen (UCPH), Denmark

**Keywords:** animal models of cancer, collagen cross-linking, lysyl oxidase, pancreatic cancer

## Abstract

Pancreatic ductal adenocarcinoma (PDAC) is one of the leading causes of cancer-related mortality. Despite significant advances made in the treatment of other cancers, current chemotherapies offer little survival benefit in this disease. Pancreaticoduodenectomy offers patients the possibility of a cure, but most will die of recurrent or metastatic disease. Hence, preventing metastatic disease in these patients would be of significant benefit. Using principal component analysis (PCA), we identified a LOX/hypoxia signature associated with poor patient survival in resectable patients. We found that LOX expression is upregulated in metastatic tumors from *Pdx1-Cre Kras*^*G12D/+*^ *Trp53*^*R172H/+*^ (KPC) mice and that inhibition of LOX in these mice suppressed metastasis. Mechanistically, LOX inhibition suppressed both migration and invasion of KPC cells. LOX inhibition also synergized with gemcitabine to kill tumors and significantly prolonged tumor-free survival in KPC mice with early-stage tumors. This was associated with stromal alterations, including increased vasculature and decreased fibrillar collagen, and increased infiltration of macrophages and neutrophils into tumors. Therefore, LOX inhibition is able to reverse many of the features that make PDAC inherently refractory to conventional therapies and targeting LOX could improve outcome in surgically resectable disease.

## Introduction

Pancreatic cancer is one of the leading causes of cancer-related death in the UK with around 7,000 cases being diagnosed every year (Mukherjee *et al*, 2008). Pancreatic ductal adenocarcinoma (PDAC) is almost universally lethal. Aggressive invasion and early metastases are characteristic of the disease, such that 80–90% of patients have surgically unresectable disease at the time of diagnosis (Giovinazzo *et al*, 2012). The majority of patients who are selected to undergo potentially curative resection for small, localized lesions almost inevitably develop recurrent or metastatic disease (Yeo *et al*, 2002), presumably due to the presence of undetected micro-metastases at initial diagnosis. Adjuvant (post-operative) chemotherapy can improve outcome despite the modest anti-tumor efficacy of these agents (Neoptolemos *et al*, 2004; Stocken *et al*, 2005). Nevertheless, overall survival remains disappointing with most patients developing local recurrence or extra-pancreatic metastasis within 2 years (Giovinazzo *et al*, 2012). Hence, there is a requirement for biomarkers to predict/prognosticate those patients that do not succumb to early recurrence post-resection and identify potentially targetable pathways that may be associated with poor prognosis. This has been made even more important by recent sequencing studies of pancreatic cancer, which have found PDAC to be very complex and contain multiple low-frequency mutations of unknown functional significance. Moreover, none of the major mutations (KRAS, TP53, SMAD4 and INK4A) currently confer treatment opportunities. Importantly, these surgically resectable patients who die of recurrent metastatic disease represent a set of patients where suppression of metastasis (either prevention or suppression of growth of micro-metastasis) may be a realistic therapeutic option and trials are underway to test whether SRC inhibitors may be beneficial in this patient set.

A characteristic feature of PDAC is the highly desmoplastic stromal microenvironment. This stroma consists of immune cells, collagen and fibronectin laid down by fibroblasts and stellate cells (Chu *et al*, 2007). In addition to contributing to disease progression, and promoting tumor growth and invasion, recent studies suggest that the stroma also limits drug delivery to tumor cells, partly explaining the profound resistance of pancreatic tumors to systemic chemotherapy agents (Olive *et al*, 2009). This situation is made worse by a very poor tumor vasculature which may also limit drug access to tumors (Olive *et al*, 2009). Consistent with this, a number of studies have shown that potential stromal markers such as S100A2 and A4 have prognostic value in patients with resected PDAC (Jamieson *et al*, 2011). More recently, there has been great interest in the role of the enzyme lysyl oxidase (LOX) in driving metastasis in epithelial cancers such as breast and colorectal cancer (Payne *et al*, 2005; Baker *et al*, 2011; Cox & Erler, 2013). LOX is a copper-dependent enzyme which cross-links collagen and elastins to drive tissue stiffness (Barker *et al*, 2012). It has previously been shown to be induced by hypoxia-inducible factor 1α (HIF1α) and can function upstream of the SRC/FAK tyrosine kinases (Payne *et al*, 2005; Erler *et al*, 2006; Baker *et al*, 2011, 2013). These studies suggest that LOX inhibition will have the potential to suppress metastasis rather than causing regression of established tumors (Cox *et al*, 2013; Cox & Erler, 2014), consistent with observations using SRC inhibitors in cancer cell lines and mouse models (Morton *et al*, 2010a). Additionally, LOX has been reported to have direct effects on cancer cells themselves through regulation of senescence (Wiel *et al*, 2013).

There is a plethora of evidence that inflammation is tumor promoting in pancreatic cancer. Pancreatitis leads to increased risk of PDAC, while cerulein promotes acinar-to-ductal metaplasia and disease progression (Guerra *et al*, 2007; Rhim *et al*, 2012). However, it is also clear that the presence of leukocytes such as neutrophils within tumors is often associated with a good prognosis in many different cancer types including pancreatic cancer (Caruso *et al*, 2002; Schaider *et al*, 2003; Jamieson *et al*, 2012). Thus far, there are no mechanistic data to explain this phenomenon, nor is it known how the desmoplastic “stiff” stroma would affect the ability of leukocytes to penetrate tumor tissue.

Given the complex interplay between the tumor and the stroma, modeling therapies in these systems require immunocompetent mice that develop tumors that closely recapitulate human disease. Genetically engineered mice carrying the common mutations that occur in PDAC rapidly generate invasive and metastatic disease (Morton *et al*, 2010b). Therefore, these can serve as excellent models in which to test therapies aimed at targeting the stroma and assess the impact of the mutations that occur in pancreatic cancer upon the stroma, invasion and metastasis. Our and others’ previous studies have shown that the accumulating mutant p53 (p53^R172H^) has gain-of-function properties over loss-of-function mutations (Olive *et al*, 2004; Jackson *et al*, 2005; Adorno *et al*, 2009; Morton *et al*, 2010b). Consistent with this, we and others have previously found that mice expressing p53^R172H^ display a far higher frequency of liver metastasis than mice with loss of p53 function (Morton *et al*, 2010b; Weissmueller *et al*, 2014). However, it has been reported that mice carrying loss of function of p53 and INK4 loss alongside *Kras*^*G12D*^ targeted to the pancreas develop adenocarcinoma that metastasizes to the liver (Bardeesy *et al*, 2006). As yet, most work has focused on how mutant p53 facilitates cell autonomous migration and invasion and has not examined whether it affects tumor–stromal interactions.

While the majority of studies had suggested that targeting the stroma may be a promising therapeutic option in this disease, recent studies have shown dramatically different results. Here, targeted sonic hedgehog depletion or myofibroblast disruption has reported decreased survival in mouse models (Lee *et al*, 2014; Ozdemir *et al*, 2014; Rhim *et al*, 2014).These studies suggest that thinking of the tumor stroma simply as a tumor-promoting entity needs to be re-evaluated and it will be very important to understand which elements of the stroma drive the chemoresistance and early progression of pancreatic cancer, and which act to constrain the tumor. It should be noted that these studies also showed that subsequent co-targeting with either anti-angiogenesis or immunotherapy could then lead to significant tumor regression.

In this study, we identify LOX, driven by mutant p53, as an important therapeutic target in pancreatic cancer, inhibition of which causes tumor necrosis in combination with gemcitabine. Importantly, the LOX–hypoxia axis defines the poor prognosis of surgically resectable cancers and can explain many of the features that contribute to making PDAC inherently refractory to conventional cytotoxic chemotherapy. Most importantly, inhibition of LOX can reverse most of these features, suggesting that this may be a promising therapeutic approach.

## Results

### LOX/hypoxia marks poor prognosis in patients with resected PDAC

**Figure 1:**
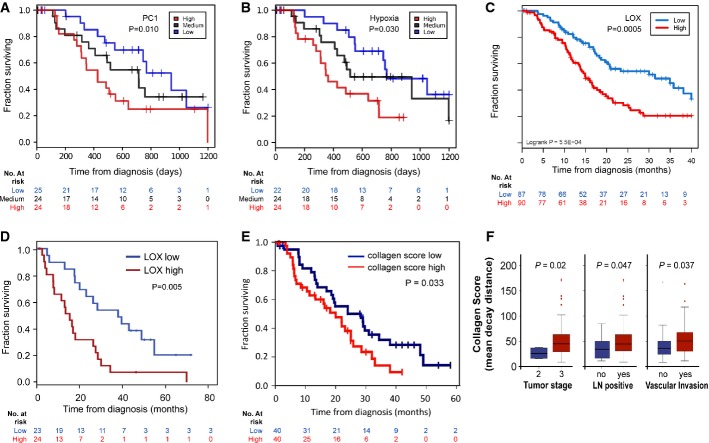
LOX/hypoxia marks poor prognosis in resectable PDAC Kaplan–Meier analysis showing cases from the Australian cohort (*n* = 73) delineated on the basis of expression of a set of hazardous genes, termed PC-1. Patients with the highest expression had a significantly poorer prognosis (red line, median survival: 11.6 months) compared with those patients with the lowest expression (blue line, median survival: 34.4 months, *P* = 0.01). The black line shows those with medium expression.

To elucidate the key components that contribute to poor prognosis in patients with PDAC, a gene signature analysis was performed on the transcriptome of 73 PDACs (Biankin *et al*, 2012). From this, a single signature was identified (called PC-1) that following cross-validation could predict survival in the discovery cohort (Fig1A). PC-1 could be split into two gene sets: a hazardous set of 321 transcripts, for which increased expression was associated with poor prognosis, and a protective set of 238 transcripts, for which increased expression was beneficial. To identify potential biological processes underlying the survival signature, we tested the hazardous and protective sets of PC-1 for overlap against the MSigDB gene set database (Subramanian *et al*, 2005). We observed that the hazardous subset of PC-1 showed significant overlap with numerous hypoxia signatures, and the protective subset was linked to lymphocyte and antigen presentation signatures (Supplementary Table S1). We confirmed that high expression of a hypoxia signature correlated with poor prognosis (Fig1B). From this hypoxia signature, we selected *LOX* for further analysis given we wished to investigate factors that could modulate the stroma that are targetable, its previous association with the mesenchymal subtype and reports of its involvement in metastasis in breast and colon cancer (Payne *et al*, 2005; Baker *et al*, 2011). We examined overall survival in terms of the expression of *LOX* using an expanded APGI cohort of 266 patients (Chou *et al*, 2013). Expression of *LOX* correlated with patient survival (Fig1C), and we confirmed this correlation using multivariate analysis (Supplementary Table S2). We next looked in our Glasgow cohort of patients where 47 patients have been profiled and once again high LOX expression correlated with a poor prognosis (Fig1D). Moreover, *LOX* was 1 out of only 5 probe sets that overlapped between the Glasgow (Jamieson *et al*, 2011) cohort and the Collisson (Collisson *et al*, 2011) signatures of poor prognosis in pancreatic cancer (the others being *S100A2, TWIST, NT5E and PAPPA*) (Supplementary Fig S1). Additionally, high expression of further LOX family members (LOXL1, 2, 3 and 4) were found to be associated with poor prognosis in the Glasgow data set (Supplementary Fig S1B–E). Taken together, and given the reproducibility across multiple patient cohorts, these findings argued for a significant role for LOX expression as a determinant of poor survival in pancreatic cancer.

Kaplan–Meier analysis showing that cases in the Australian cohort that fell in the highest quartile of a hypoxia signature (red line, median survival: 11.4 months) have significantly decreased survival compared with those in the lowest quartile (blue line, median survival: 25.2 months; *P* = 0.03).

Kaplan–Meier analysis showing that cases in the APGI cohort (*n* = 266) with high *LOX* expression above the 3^rd^ quantile (red line) have significantly decreased survival compared to those with low expression below the 1^st^ quantile (blue line).

Kaplan–Meier analysis showing that cases in the Glasgow cohort with high *LOX* expression (red line, *n* = 24) have significantly decreased survival compared with those with low expression (blue line, *n* = 23; *P* = 0.005).

Kaplan–Meier analysis showing that fibrillar collagen is significantly associated with reduced survival in human PDAC (20 months vs. 28.3 months, *P* = 0.033).

Mean decay distance of the second harmonic generation (SHG) signal emitted by human PDAC-associated collagen. Mean decay distance is represented by boxplots showing the second and third quartile of the data with the whiskers indicating the maximum and minimum data points. Outliers are indicated by individual markers. Stage T3 tumors have a higher collagen mean decay distance score compared with Stage T2 tumors (*P* = 0.02). Lymph node-positive tumors have a higher collagen mean decay distance score vs. lymph node-negative tumors (*P* = 0.047). Tumors showing vascular invasion have a higher collagen mean decay distance score than non-vascular invasive tumors (*P* = 0.037).

As LOX is associated with regulation of collagen cross-linking, we assessed the status of fibrillar collagen in human PDAC. Using multiphoton microscopy, we analyzed the second harmonic resonance signal of collagen in a human pancreatic tissue microarray (TMA) consisting of 80 patients. Second harmonic generation (SHG) imaging of collagen has been used previously to identify collagen “signatures” in mammary tumors, which may impact on the ability of cancer cell migration (Provenzano *et al*, 2006, 2009). In our study, we found that fibrillar collagen was significantly associated with reduced survival (20 months vs. 28.3 months, *P* = 0.033) (Fig1E). Further, we showed that fibrillar collagen was significantly associated with increased tumor stage, lymph node spread and vascular invasion (Fig1F). This suggested that the generation of fibrillar collagen may be important in PDAC disease progression and that the inhibition of cross-linking needed to generate collagen fibers via LOX inhibition may be important therapeutically.

**Figure 2:**
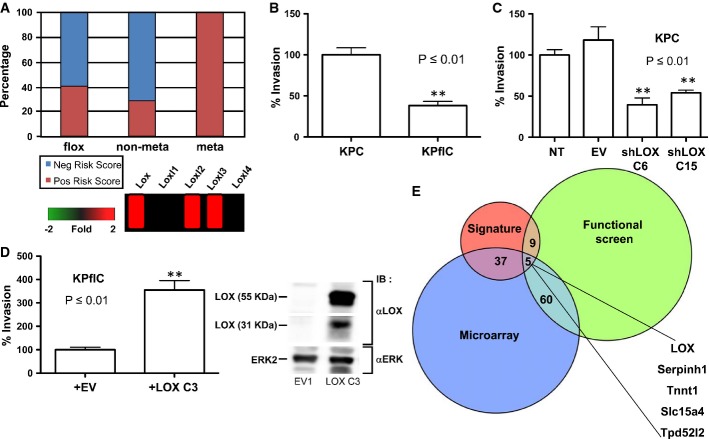
LOX expression is required for invasion in a mutant p53-driven model of PDAC PC-1 signature is predictive of metastatic disease in mouse models of pancreatic cancer. Log-transformed expressions of signature transcripts from mouse tumor microarrays were mean-centered across samples and scaled to unit variance. These values were then multiplied by the matching loading values from the PC-1 signature and summarized for each sample across all transcripts to yield the risk score for that sample.

In order to further investigate this, we used immunocompetent, autochthonous genetically engineered mouse models of PDAC, which are initiated by targeting *Kras* mutation to the murine pancreas using the *Pdx1-Cre* transgene. Without the addition of cooperating mutations, only one-third of *Pdx1-Cre Kras*^*G12D/+*^ mice develop PDAC by 500 days (Hingorani *et al*, 2003). However, when p53 is additionally targeted by deletion or mutation, mice rapidly develop invasive adenocarcinoma with a median latency of between 120 and 180 days (Hingorani *et al*, 2005) and we have previously found that p53 mutation, but not loss, could drive metastasis in this model (Morton *et al*, 2010b). In order to characterize the gene expression changes that underlie metastatic disease, we carried out microarray analysis of tumors driven by *Kras* mutation and mutation of one copy of p53 or deletion of p53. To address whether transcriptional changes observed in pancreatic cancer patients were reflected in our mouse models, we applied loading values obtained from the PC-1 human tumor signature to our mouse microarray data sets (http://www.ncbi.nlm.nih.gov/geo/query/acc.cgi?acc=GSE67358). This allowed us to derive risk scores for each individual mouse transcriptome. While the majority of mice that had non-metastatic tumors had negative risk scores, 100% of KPC mice that developed metastatic disease had positive risk scores (Fig2A, top). Of the gene expression changes observed between metastatic and non-metastatic tumors, multiple members of the LOX family were overexpressed in metastatic disease (Fig2A, bottom).

Inverted invasion assays were performed with PDAC tumor cell lines from KPC and KP^fl^C mice. Tumor cell lines bearing mutant p53^R172H^ (KPC) invade significantly further than tumor cells with deletion of 1 copy of p53 (KP^fl^C) (*P* ≤ 0.01). Data are shown as the average of four wells + SEM.

Introduction of shRNA targeting Lox into KPC tumor cells significantly inhibits invasion (*P* ≤ 0.01 by unpaired Student’s *t*-test). Data are shown as the average of four wells + SEM.

Introduction of exogenous LOX into KP^fl^C tumor cells significantly promotes invasion (left panel, *P* ≤ 0.01 by unpaired Student’s *t*-test). LOX expression was assessed by immunoblotting (right panel). Columns indicate the mean of four well and error bars indicate SEM.

Integration of heterogeneous data sets identifies LOX as a therapeutic target in pancreatic cancer. Components of the hazardous PC-1 signature are overlaid with genes found to be overexpressed in a microarray and hits that cause a reduction in viable cell number in an RNAi functional screen. Overlap is shown as a proportional Venn diagram. Source data are available online for this figure.

### LOX is required for mutant p53-driven invasion

Given that our data suggest a role for LOX in mutant p53-driven metastatic PDAC, we decided to address the consequences for cell migration and invasion, of manipulating LOX expression in PDAC cell models. For this, we used both KPC cell lines and cells derived from *Pdx1-Cre Kras*^*G12D/+*^ *p53*^*flox/+*^ (KP^fl^C) tumors. Initially, to assess whether LOX expression was required for invasion, we used siRNA to knockdown LOX expression and measured migration of KPC cells through a Matrigel matrix (Supplementary Fig S2A). Loss of LOX expression significantly impaired KPC cell migration, and this could be rescued by recombinant LOX protein. Inhibition of LOX in KPC cells led to a reduction in SRC phosphorylation (Supplementary Fig S2B), and we confirmed that low doses of the SRC inhibitor dasatinib resulted in a slowing of wound healing (Supplementary Fig S2C). Given our previous data showing a requirement for SRC in KPC cell invasion (Morton *et al*, 2010a), this suggests that the pro-migratory effects of LOX could be mediated at least in part by SRC activation. We confirmed that LOX knockdown in human Panc-1 cells had similar effects on migration (Supplementary Fig S2D). We have previously shown that the ability of mutant p53 cells (and not p53-deleted cells) to invade *in vitro* correlates well with the ability of these cells to metastasize *in vivo* (Fig2B). Analysis of the expression of LOX family members in these cell lines showed a clear increase in the expression of LOX and LOX family members in cell lines carrying mutant but not loss-of-function p53, consistent with our microarray analyses (Supplementary Fig S2E). Thus, we carried out stable knockdown of LOX in KPC cells using shRNA and found this significantly reduced invasion of mutant p53 cells (Fig2C; Supplementary Fig S2F). Conversely, overexpression of LOX in p53 loss-of-function tumor cells promoted invasion of these cells (Fig2D).

In order to determine the effects of the modulation of LOX expression in these cell models *in vivo,* we carried out a series of allograft experiments. LOX knockdown slowed allograft growth of KPC cells (Supplementary Fig S2G), while LOX overexpression resulted in faster growth of KP^fl^C cells (Supplementary Fig S2H).

In parallel, we also performed a functional screen on primary cells from *Pdx1-Cre Kras*^*G12D*^ *p53*^*R172H*^ (KPC) metastatic tumors. Overall, we found that 920 siRNAs reduced cell viability using a Z-score threshold of -3 (Supplementary Table S3). A number of these screen hits were extracellular matrix components, including LOX, fibronectin and tenascin C. To enhance the predictive power of our results, we overlaid the siRNA data with microarray data and components of the hazardous PC-1 signature. We identified 106 hits using two of the approaches, and five that were hits in all three analyses, one of which was LOX (Fig2E). In order to confirm the results of the screen, we knocked *Lox* down, observed a reduction in the number of viable cells (Supplementary Fig S3A) and confirmed that this effect could be rescued by recombinant protein (Supplementary Fig S3B). Quantitation of live and dead cell numbers by trypan blue exclusion showed that while *Lox* knockdown led to a decrease in live cell number, this was not associated with an increase in cell death (Supplementary Fig S3C). In response to *Lox* knockdown, KPC cells expressed higher levels of fibronectin mRNA and we found that the effects of *Lox* knockdown could be enhanced by blocking integrin/fibronectin interaction using an α5-integrin-blocking antibody (Supplementary Fig S3D). Thus, LOX inhibition may also have impacts on tumor cells themselves that are mediated by integrin and fibronectin. This suggests that in contrast to the potentially oncogenic role of total depletion of tumor myofibroblasts (Ozdemir *et al*, 2014), blockade of collagen cross-linking and LOX should not have tumor-promoting effects. These data from cell models suggest that targeting LOX has the potential to inhibit both cell invasion and growth at the secondary site, suggesting a potential approach to target metastasis *in vivo*.

### Treatment of KPC mice with LOX-blocking antibody in combination with gemcitabine strongly suppresses tumorigenesis

**Figure 3:**
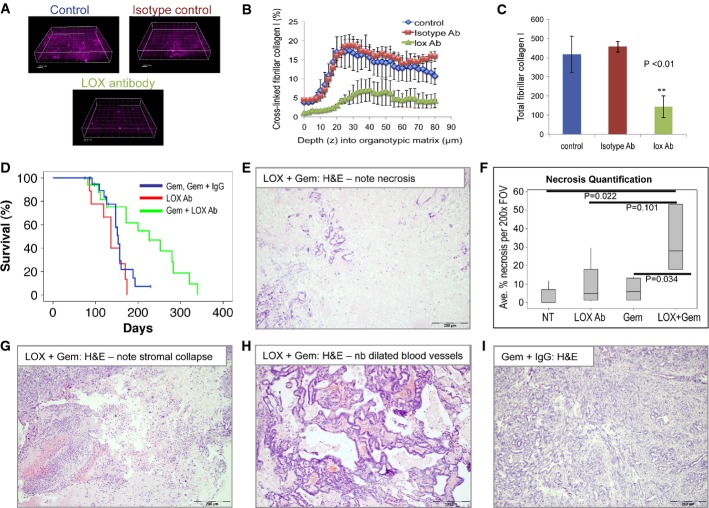
LOX inhibition, in combination with gemcitabine, extends pancreatic cancer-free survival in KPC mice A Representative fluorescence images showing the SHG signal from cross-linked collagen in untreated, LOX antibody-treated or isotype control-treated organotypic cultures of primary fibroblasts and rat tail fibrillar collagen I.

Thus far, all of our functional experiments with LOX inhibition were not performed in any of the autochthonous models of PDAC that faithfully recapitulate the human disease. Our prediction was that in this context, LOX inhibition was likely to have more of an impact, as PDAC is well known to be a highly desmoplastic tumor with marked levels of stromal collagen. Thus, in this environment, expression of LOX protein should have a marked impact on the surrounding stroma, driving the cross-linking of collagen and elastin in the tumor microenvironment. To investigate the significance of LOX overexpression in PDAC, we used a LOX function-blocking antibody (Erler *et al*, 2006, 2009; Baker *et al*, 2011, 2013; Cox *et al*, 2013). This antibody is specific to LOX and does not recognize the other members of the LOX family in Western blotting, while peptide blocking studies and protein binding studies found no evidence of binding to LOXL2 (Le *et al*, 2009; Baker *et al*, 2011, 2013; Cox *et al*, 2013). First, to justify that the antibody could modify LOX activity, we investigated the ability of the antibody to block collagen cross-linking in an *ex vivo* organotypic assay. In this assay, rat tail collagen I is mixed with fibroblasts that secrete LOX protein. The fibroblasts cross-link and contract the collagen and produce a matrix that is often used to measure invasion *ex vivo* (Timpson *et al*, 2011a,b). Addition of the LOX-blocking antibody, but not the isotype control antibody, to these cultures markedly reduced fibrillar collagen (Fig3A–C). Taken together with previous work using this antibody, our data support the ability of this strategy to block LOX activity.

B Chart showing the percentage of cross-linked collagen by depth, based on SHG signal, in organotypic matrices treated as indicated. Error bars indicate SEM.

C Barchart showing total cross-linked collagen in organotypic matrices treated as indicated. The experiment was performed in triplicate with three different zones imaged for 80 μm, at 2.5-μm sections each. Data are shown as total pixels ± SEM.

D Kaplan–Meier (non-parametric) survival curve showing significantly extended tumor-free survival of KPC mice treated with LOX antibody (1 mg/kg i.p., twice weekly) + gemcitabine (100 mg/kg i.p., twice weekly) (green line, *n* = 17), compared with gemcitabine ± isotype control antibody-treated mice (blue line, *n* = 22, *P* = 0.014) or LOX antibody alone (red line, *n* = 9). Treatment was initiated when mice were 70 days old (randomization was not used when recruiting the mice) and the mice were treated twice weekly. Censored mice did not develop PDAC.

E H&E-stained section of PDAC harvested from LOX + gemcitabine-treated KPC mouse. Note necrosis.

F Boxplot showing quantification of necrosis in H&E sections of PDACs harvested from these mice, as indicated. At least 30 fields of view from at least five mice per cohort were scored.

G–H H&E-stained sections of PDAC harvested from LOX + gemcitabine-treated KPC mice. Note stromal collapse (G) and marked dilation of blood vessels (H).

I Representative H&E image showing a section of PDAC harvested from a control-treated KPC mouse.

To assess the functional importance of LOX *in vivo*, we next treated KPC mice from day 70 with LOX-blocking antibody, gemcitabine, isotype control antibody with gemcitabine and LOX-blocking antibody with gemcitabine. We chose this timepoint for these studies since our aim is to mimic surgically resectable disease without evidence of overt, macroscopic metastases. Moreover, in the recent study using hedgehog inhibitors showed that treatment at a slightly earlier stage of disease revealed the tumor-promoting effects of hedgehog inhibition which were not seen in mice with late-stage disease (Rhim *et al*, 2014). This is important as the human hedgehog inhibitor trials stopped due to concerns over disease acceleration. At this 70-day timepoint, mice have widespread advanced pancreatic neoplasia (Hingorani *et al*, 2005; Morton *et al*, 2010a). Cohort size was based on our previously reported work where we have shown that treatment with dasatinib at this timepoint reduced metastasis without affecting primary tumor growth (Morton *et al*, 2010a). In addition, tumors in these mice are resistant to gemcitabine treatment. This contrasts with chemoprevention experiments we have conducted where gemcitabine treatment from 5 to 6 weeks suppresses pancreatic tumorigenesis (Supplementary Fig S4A).

In comparison with previous cohorts and control-treated KPC mice, we saw a significant slowing of tumorigenesis in mice treated with the combination of LOX antibody and gemcitabine, with a median survival of 226 days, compared with 153 days in mice treated with either gemcitabine or gemcitabine + isotype control antibody (Fig3D, Table1, *P* = 0.014). Interestingly, symptoms of drug toxicity due to long-term gemcitabine treatment meant that six of these mice were free of pancreatic tumors at the time of sacrifice, including two mice that were > 200 days at the time of sacrifice. Moreover, a significant reduction in metastasis formation was observed in LOX + gemcitabine-treated mice (2/13 mice), while a complete absence of metastasis was observed in the mice treated with LOX-blocking antibody (Table1). Similar to previous studies, mice treated with gemcitabine alone or gemcitabine + isotype control antibody developed metastasis in 80% of cases (Table1). As we have previously observed (Morton *et al*, 2010a), an absence of metastasis alone was not sufficient to increase survival in the LOX-treated mice as they still became sick of primary tumor burden.

**Table 1:** Metastatic burden in LOX Ab-treated PDAC-bearing mice

Treatment	Median survival	Metastatic burden				
		Metastases	Liver	Lung	Diaphragm	Peritoneal
Gem	126 days	3/3	2/3	0/3	1/3	1/3
IgG + Gem	153 days	8/11	5/11	1/11	1/11	1/11
LOX Ab	136 days	0/8	0/8	0/8	0/8	0/8
LOX + Gem	226 days	2/13	2/13	2/13	2/13	0/13

Table showing metastatic burden in PDAC-bearing mice treated as indicated. LOX Ab vs. Gem, *P* = 0.0106 (Chi^2^ + Yates’ correction); LOX + Gem vs. IgG + Gem, *P* = 0.0154 (Chi^2^ + Yates correction). LOX, lysyl oxidase; Ab, antibody; Gem, gemcitabine.

**Figure 4:**
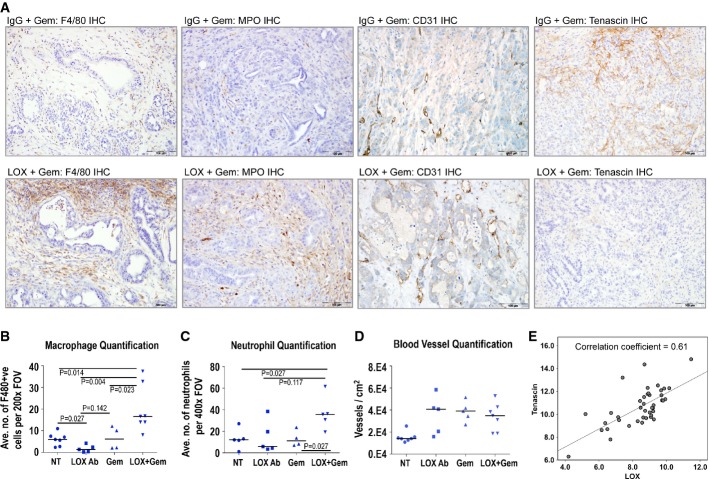
Enhanced leukocyte infiltration and stromal markers in LOX antibody-treated mice A Outer left panels: Immunohistochemical analysis of macrophage infiltration (by F4/80 staining) in tumors from isotype control + gemcitabine-treated KPC mice vs. LOX antibody + gemcitabine-treated KPC mice. Inner left panels: Immunohistochemical analysis of neutrophil infiltration (by MPO staining) in tumors from isotype control + gemcitabine-treated KPC mice vs. LOX antibody + gemcitabine-treated KPC mice. Inner right panels: Immunohistochemical analysis of tumor vasculature (by CD31 staining) in tumors from isotype control + gemcitabine-treated KPC mice vs. LOX antibody + gemcitabine-treated KPC mice. Outer right panels: Immunohistochemical analysis of tenascin C expression in tumors from isotype control + gemcitabine-treated KPC mice vs. LOX antibody + gemcitabine-treated KPC mice.

To understand the mechanism of action of the LOX-blocking antibody and the increased benefit with gemcitabine, we next analyzed the tumor and stroma from these mice. Histological examination of these tumors revealed a marked and significant increase in necrosis in the tumors treated with the combination of LOX-blocking antibody and gemcitabine, along with stromal changes and dilation of blood vessels (Fig3E–H). In contrast, virtually no necrosis was observed in control tumors (Fig3I). Moreover, a marked leukocyte infiltration was also observed and immunohistochemical staining for both macrophages (by F4/80 IHC, left panels Fig4A, quantitation Fig4B) and neutrophils (MPO IHC, 2^nd^ from left panels Fig4A, quantitation Fig4C). Another hallmark of tumors arising in the KPC model is a poor vasculature and this was again subverted, with a clearly increased number of “open” blood vessels in the KPC mice treated with LOX-blocking antibody ± gemcitabine (CD31 IHC, 2^nd^ from right panels Fig4A), although interestingly, vessel number was elevated in all treatment groups (Fig4D). Tenascin C is a stromal marker previously reported to be induced in pancreatic cancer and has been implicated in metastasis in other cancer models (Juuti *et al*, 2004; Oskarsson *et al*, 2011). We therefore examined whether there was a correlation between LOX inhibition and tenascin C expression and found a complete absence of stromal staining in LOX antibody + gemcitabine-treated mice (Fig4A right panels). Moreover, when we further examined the specific relationships between stromal markers such as LOX and tenascin C, we found a significant correlation in human PDAC (Fig4E). Interestingly, there was no significant difference in levels of proliferation, or apoptosis (Supplementary Fig S4B and C) following treatment with LOX, gemcitabine or LOX and gemcitabine combination. This suggested that although LOX inhibition can have an effect on the tumor cells in culture, this was not sufficient to affect tumor proliferation *in vivo*. As one of the consequence of LOX knockdown *in vitro* was an upregulation of fibronectin (Supplementary Fig S3D), we tested the effect of further increases in fibronectin and found this could rescue the inhibition of proliferation caused by LOX inhibition (Supplementary Fig S4D). *In vivo*, KPC tumors have very high levels of fibronectin (Supplementary Fig S4E).

B–D Plots showing quantification of macrophage infiltration (B), neutrophil infiltration (C) and tumor vasculature (D) in tumors from KPC mice treated as indicated. At least 30 fields of view from at least four mice per cohort were scored, and scoring was conducted blind. *P*-values were calculated using Mann–Whitney *U*-test and median is indicated by horizontal lines.

E Correlation of LOX protein with tenascin C expression in 47 cases of PDAC (Spearman’s rho correlation coefficient = 0.61; *P *<* *0.0001).

Importantly, when we performed picrosirius red staining to examine the quantity of collagen in tumors from mice treated with isotype control antibody, LOX antibody alone or in combination with gemcitabine, we did not observe any significant differences (Supplementary Fig S5A and B). However, when we examined the degree of fibrillar collagen by SHG, as performed on the human tissue microarray earlier, we found that tumors in mice treated with LOX-blocking antibody + gemcitabine had significantly less fibrillar collagen when compared with mice treated with gemcitabine alone (Supplementary Fig S5A and C).

Interestingly, we saw no evidence of increased gemcitabine penetrance into the tumor when mice were treated with LOX antibody and gemcitabine compared with isotype control and gemcitabine (Supplementary Table S4). Thus, combining LOX Ab with gemcitabine does not improve survival by increasing drug penetration. Instead, we would suggest that the effects on the stroma result in the loss of key microenvironment-mediated pro-survival signals that would normally drive resistance to therapy. We also observed that KPC cells grown on collagen were less sensitive to gemcitabine treatment than those grown on an uncoated plate (Supplementary Fig S5D). Taken together, our data clearly show that the LOX-blocking antibody, when administered in combination with gemcitabine, can reverse many of the hallmarks of PDAC that are thought to yield resistance to treatment and most pertinently here, it can reverse the two pathways that are conferring poor prognosis in PDAC (hypoxia/LOX).

### LOX inhibition at late-stage disease

Mechanistically, our data were suggestive that LOX-mediated collagen cross-linking is a pro-survival and metastatic factor in PDAC. If this was the case, one would predict that LOX inhibition would have a more marginal effect in late-stage disease due to tumours already having significant levels of cross-linked collagen. Therefore, we treated mice with a palpable tumor burden with the LOX-blocking antibody alone and in combination with gemcitabine.

As predicted, treatment with LOX-blocking antibody and gemcitabine did not have a significant effect on survival with mice with late-stage disease, although fewer of these mice had metastasis following treatment (Supplementary Fig S6A). We also did not observe alterations of gemcitabine uptake into the tumor or changes in collagen levels (Supplementary Fig S6B and D). Importantly, many of the alterations we observe in mice treated from an earlier stage were not recapitulated in mice with late-stage disease; namely, no significant changes were found in blood vessel number, necrosis, tenascin C levels or macrophage infiltration (Supplementary Fig S6C and E–G). We did observe alterations in immune cell infiltration with decreased neutrophil infiltration and increased T-cell infiltration (Supplementary Fig S6H and I). These data are consistent with our model that anti-LOX therapies work by modulating collagen cross-linking.

## Discussion

Pancreatic ductal adenocarcinoma has a dire prognosis with 85% of patients presenting with tumors that are unresectable. The current standard of care therapy for these patients only prolongs survival to a median of 6 months. Even patients that undergo potentially curative resection almost inevitably develop metastatic disease. Thus, new treatment options are required both for patients with resectable disease and for patients with advanced disease**.**

In this study, we show that a functional axis can define survival in patients that undergo pancreaticoduodenectomy and that a “hypoxia/LOX” signature and high levels of fibrillar collagen are associated with poor prognosis. This suggests that fibrillar collagen (potentially quantifiable by elastography using endoscope-guided ultrasound in the clinic) and LOX expression have potential as prognostic markers in pancreatic cancer. We also show that manipulation of a key element of this hypoxia/LOX signature can reverse most of the features of the axis and leads to a remarkable increase in survival of KPC mice. This model can be seen a gold standard for pancreatic cancer treatment, as it recapitulates many of the clinical features of the human disease including its poor response to gemcitabine (and most other cytotoxic chemotherapy agents), and mice develop invasive and metastatic disease.

One striking feature of LOX inhibition in the KPC model was stromal alterations that were associated with increased tumor vascularization, immune cell infiltration and drug efficacy. Recently, there has been considerable interest in the role of tumor–stromal interaction in pancreatic cancer. Previous studies showed that targeting the stroma with inhibitors of Shh signaling led to increased (Olive *et al*, 2009) or decreased (Rhim *et al*, 2014) survival in PDAC mouse models, depending on the treatment regime and mouse model used. A further study found that targeting stroma through myofibroblast deletion also led to decreased survival (Ozdemir *et al*, 2014). Stromal alterations have been associated with increased vascularization of tumors (Olive *et al*, 2009; Rhim *et al*, 2014) and increased efficacy of gemcitabine (Olive *et al*, 2009) and anti-angiogenic therapies (Rhim *et al*, 2014). For this reason, and in order to mimic surgically resectable disease without overt metastases, we commenced treatment of mice aged 70 days. Here, we saw no tumor acceleration at any stage by LOX inhibition. It is interesting to note that in the myofibroblast study, despite depletion of the myofibroblasts, neither LOX levels nor collagen cross-linking was altered. This fits well with our data where tumor cell secretes LOX and suggests it is the cross-linking of collagen that is critical in defining the pro-tumorigenic quality of the stromal. Thus, it becomes very important for the community to ascertain the precise pro-tumorigenic elements of the stroma and whether these are targetable, and in which patients, as stromal targeting agents may affect tumor dynamics in different ways depending on the tumor stage at diagnosis.

One of the challenges of the literature is the low number of prognostic studies for which any overlap exists. One of the strengths of the analyses presented here is that LOX is an indicator of poor prognosis in both the Glasgow and Australian studies, as well as the Collisson patient cohort (Collisson *et al*, 2011). Moreover, while specific immune cell infiltration has been suggestive of a poor prognosis (Mitchem *et al*, 2013), leukocyte infiltration *per se* can confer a good prognosis in PDAC and in other cancer types (Caruso *et al*, 2002; Jamieson *et al*, 2012). One of the questions raised from our work is “What is the direct relationship between LOX and immune cell infiltration?” and we propose a model in which a tightly cross-linked collagen matrix could block immune cell penetration into tumors or inhibit chemotaxis. Importantly, loss of myofibroblasts in PDAC was also shown to allow T-cell entry and immunotherapy (Ozdemir *et al*, 2014). Consistent with this, we observe increased T-cell infiltration into tumors during short-term treatment with anti-LOX therapy, while macrophage and neutrophil infiltration was a feature of longer-term treatment. Thus, future studies examining combinatorial work with LOX inhibition and immunotherapy might produce even more striking results.

Poor vascularization and “poor quality” vascularization in PDAC have been shown previously and may be the predominant cause of the hypoxia signature. There is a well-established link between hypoxia and upregulation of LOX expression (Erler *et al*, 2006), which only reinforces this situation and further impedes drug access to tumors, although it has also previously been shown that LOX activity can stimulate angiogenesis in colon cancer (Baker *et al*, 2013). One other key suggestion from this work is that this prognostic signature could become a predictive signature to select patients falling within the poor component of the axis and who would most benefit from treatment with putative LOX inhibitors.

Our work may also yield new insights into how mutant p53 may drive metastasis in PDAC. Our previous studies have shown key roles for mutant p53 in driving the mechanics of invasion and migration (integrin and met recycling) (Muller *et al*, 2009, 2013). Here we show that mutant p53 within a tumor cell may also shape the external tumor microenvironment. This may help to explain our previous observations that mutant p53, but not loss-of-function p53, leads to PDAC metastasis in this mouse model (Morton *et al*, 2010b). The idea that mutant p53 in tumor cells may help shape their microenvironment also aligns with our previous work which found that RhoA activity was localized at the tips of mutant p53-expressing pancreatic cancer cells during invasion (Timpson *et al*, 2011b). Mechanistically, this may also be due in part to a reduction in SRC activity, which we have previously shown to be important for metastasis in this system and has been shown to be downstream of LOX in many other systems (Morton *et al*, 2010a). Indeed, we previously showed that LOX signals through SRC to increase colon cancer tumor cell proliferation, invasion and metastasis (Baker *et al*, 2011). It is interesting to note that *in vitro*, we also observe cell-intrinsic anti-proliferative effects of LOX knockdown. This may explain the suppression of metastasis in mice treated with LOX antibody alone. Cells in culture may be subjected to similar stresses as metastasizing tumor cells, and in this situation, cell matrix proteins such as LOX could support cell survival and proliferation.

Very recently, it has been announced that LOXL2-blocking antibodies did not improve outcome in a phase two trial in advanced pancreatic cancer (Benson *et al*, 2014). This is consistent with our data as the LOX-blocking antibody was unable to suppress pancreatic cancer in mice with advanced disease. This correlated with the inability of the antibody to reverse the collagen cross-linking at this stage. It should be noted that to our knowledge experiments with LOXL2 antibodies have not been performed in autochthonous models and it would be of interest to see whether LOXL2-blocking antibodies would suppress metastasis.

In summary, we believe we have identified a crucial axis in PDAC that could be exploited as a therapeutic target. This would strongly support further development of LOX inhibitors for PDAC and the continued investigation of other approaches to target the tumor microenvironment in this disease. We propose that LOX inhibitors could potentially improve disease-free and overall survival when administered in combination with gemcitabine (standard of care) following resection of PDAC in selected patients, and could potentially enhance the efficacy of gemcitabine-containing chemotherapy regimens in patients with advanced, inoperable disease, thereby improving objective responses and overall survival.

## Materials and Methods

### Identification of a hypoxia-related survival signature

RNA of bulk primary tumor specimens from 73 patients with confirmed PDAC was arrayed on Illumina HumanHT-12 v4 microarrays (http://www.ncbi.nlm.nih.gov/geo/query/acc.cgi?acc=GSE50827). Samples were from patients recruited into the Australian Pancreatic Cancer Genome Initiative (Biankin *et al*, 2012). Arrays were processed using the R package lumi (R version 2.15.1, lumi version 2.8.0) with VST transformation and RSN normalization, and batch effects were removed using ComBat (Johnson *et al*, 2007). Probes were variance-filtered, collapsed into per-gene transcripts by selecting the highest variance probe for each gene and filtered for univariate survival association using a 1,000,000-round permutation test based on the FAST statistic (Gorst-Rasmussen & Scheike, 2012). Transcripts were selected if their local FDR as estimated by the Strimmer method (Strimmer, 2008) was less than 0.15. The expression of survival-associated transcripts was decomposed into metagenes by PCA, and the ability of each principal component to predict survival was assessed based on the values calculated by leave-one-out cross-validation applied to the full metagene discovery pipeline. Gene set over-representation analysis was performed using hypergeometric tests against MSigDB classes c1, c2 and c3; sets were called significant if their Benjamini–Yekutieli FDR-corrected *P*-values (Benjamini & Yekutieli, 2001) were less than 0.05.

### Scoring gene expression data for a hypoxia signature

A hypoxia meta-signature was generated by first selecting MSigDB v3.0 c2 gene sets with names containing the term “HYPOXIA.” Twenty-two gene sets were identified, containing 1,377 genes. Fifty-five genes were represented in more than 20% of the hypoxia sets and formed a hypoxia meta-signature. Samples from the APGI microarray cohort were scored on this meta-signature by PCA: Mean-centered gene expression measurements for only the meta-signature genes were fit by PCA, and the loadings of the first principal component were used as hypoxia scores. To link increased values of the hypoxia score with increased hypoxia, scores were inverted if necessary, so that the rotation of VEGFA mRNA, a known hypoxia-induced transcript present in the meta-signature, was positive.

### Glasgow cohort gene expression analysis

Macrodissected fresh-frozen pancreatic tissue samples were used: 47 PDACs were obtained from patients undergoing pancreaticoduodenectomy at the West of Scotland Pancreatic Unit. Tissue was collected prospectively with local ethical approval, fully informed consent, pathology assessment and validation. Only histologically proven PDACs were included. Complete clinicopathological, follow-up and recurrence data were available. RNA was isolated from frozen tissue by standard TRIzol (Invitrogen) extraction according to the manufacturer’s instructions. A nanodrop spectrophotometer (NanoDrop Tech) quantified total RNA, whereas purity and integrity was assessed on the Agilent 2100 Bioanalyzer with the RNA 6000 Nano LabChipVR reagent set (Agilent Technologies). Samples with a RNA integrity number (RIN) above 7.0 were deemed suitable for downstream analysis. Kaplan–Meier survival analysis was used to analyze overall survival from the time of surgery. A log-rank test was used to compare the length of survival between curves. Statistical significance was set at a *P*-value of < 0.05. All statistical analyses were performed using SPSS version 19.0 (version 19.0. Armonk, NY: IBM Corp.)

### KPC cell transfection and high-throughput functional screens

Generation of KPC and KPflC primary cell models from transgenic mice has previously been described (Morton *et al*, 2010b). Cells were confirmed to be mycoplasma-free using an in-house mycoplasma testing service. All siRNAs were siGENOME smartpools and were used at a concentration of 25 nM (Thermo Fisher Scientific). Transfection of KPC cells was carried out with Dreamfect Gold (Oz Bioscience), and transfection of Panc-1 cells was carried out with Lipofectamine 2000 (Qiagen). Cell viability was measured by the absorbance 72 h after transfection using Cell Titer Blue (Promega).

Lox expression was measured by quantitative RT–PCR (forward primer: TCTTCTGCTGCGTGACAACC, reverse primer: GAGAAACCAGCTTGGAACCAG) using GAPDH expression as a control (forward primer: ACATCAAGAAGGTGGTGAAGCAGG, reverse primer: CCCTGTTGCTGTAGCCGTATT C).

The following antibodies were used at the indicated concentrations: LOX (Millipore ABT112, 1 in 500 dilution), SRC (Cancer Research UK, 1 in 1,000 dilution) and YES1 pY537/SRC pY530 (Abgent AP3254a, 1 in 500 dilution). The α5-integrin-blocking antibody (mAb16) has been previously described (Muller *et al*, 2009). Recombinant LOX protein was purchased from Cambridge Bioscience (Origene TP313323).

For functional screens, 72 h following transfection, 50 μM resazurin (Sigma) was added to the culture medium. Fluorescence was measured using an Envision plate reader (Perkin Elmer).

### Matrigel migration assays

Following 48 h transfection, wounds were made in the cell monolayer using a 96-well wound*-*making tool (Essen Bioscience). The wounds were then overlaid with Matrigel and cell migration was monitored using an Incucyte system (Essen Biosciences).

### shLox and LOX overexpression in PDAC cells

KPC cells were transfected with 1 μg of MISSION® pLKO.1-puro empty vector control plasmid DNA (EV) or short hairpin targeting LOX-containing plasmid (shLOX) (Sigma) using Lipofectamine (Invitrogen) for 48 h. Cells were then transferred into 2.5 μg/ml puromycin (Invitrogen) containing medium for single-cell clone selection. Two representative clones (shLOX C6 and shLOX C15) were then chosen for further characterization.

KP^fl^C cells were transfected with 1 μg of pCEF empty vector control plasmid DNA (EV) or pCEF full-length LOX-containing plasmid (LOX H1) using Lipofectamine (Invitrogen) for 48 h. Cells were then transferred into 1 mg/ml G418 (Invitrogen) medium for single-cell clone selection. One representative clone (LOX H1) was chosen for further characterization. Similarly, one additional KP^fl^C overexpressing LOX clone and corresponding empty vector control were generated by co-transfecting KP^fl^C cells with 1 μg of pLINK empty vector control plasmid DNA (EV) or pLINK full-length LOX-containing plasmid (LOX C3) plus 0.1 1 μg of PCDNA plasmid in order to confer cells the hygromycin resistance. After 48 h, transfection cells were then transferred into 0.5 mg/ml hygromycin (Invitrogen) medium for single-cell clone selection.

Inverted *in vitro* invasion assays were carried out as previously described (Arozarena *et al*, 2011).

### Determination of collagen quantity and quality

Collagen second harmonic images were collected using a LaVision Biotec Trim-scope equipped with a Coherent Chameleon Ti:Sapphire femtosecond pulsed laser. An excitation wavelength of 890 nm was used so that the SHG would be generated at a central wavelength of 445 nm and focused to the sample plane by a long working distance of 20× (NA = 0.95) water immersion objective from Olympus. A 15-μm-deep stack was imaged over a region of 500 μm by 500 μm, with at least six regions of interest per slide taken. Image analysis was performed using ImageJ. To calculate the total area occupied by collagen, a macro was written to let the user select a directory containing the raw OME-TIFF stacks from the collagen SHG channel and these were loaded in order and a maximum projection intensity image was produced. An area calculator plugin was then run which gave as the output the percentage area covered. A similar technique was developed for performing the GLCM texture analysis. Firstly, the user selected a directory containing the collagen stack images. A maximum projection image was then produced and duplicated. The duplicate image was then automatically thresholded to produce a mask that was then applied to the original maximum projection image. This removed the background noise bias introduced in the GLCM analysis by selecting only the collagen SHG signal. The masked image was then passed to the GLCM texture plugin, which had been modified so that it could be operated in a nested loop for varying pixel comparison distances and alternative directions of comparison. The output of the plugin for each image was 100 rows of the five texture parameters over each of four directions, so in total 2,000 parameter values. These were saved as a text data file for each image. When all the images in the directory were analyzed, the data files were processed using a Matlab script that eventually gave the mean of each texture parameter for each image. These were then imported into Prism, where a double-exponential decay model was fit to the data and the weighted mean decay distance for each sample was calculated in Excel.

### Glasgow cohort tissue microarray analysis

All patients gave written, informed consent for the collection of tissue samples, and approval for collection was obtained from the local research ethics committee. All cases underwent a standardized pancreaticoduodenectomy. A total of 400 cores from a total of 80 PDAC resections with a full spectrum of clinicopathological features were arrayed. At least five tumor cores (0.6 mm) and two from adjacent normal tissue were sampled. Complete follow-up data were available for all cases. Boxplots were used to show collagen score in stage 2 vs. stage 3 tumors, lymph node-positive and lymph node-negative tumors, and vascular invasion-positive and vascular invasion-negative tumors, with these groups compared statistically using a Mann–Whitney *U*-test. Kaplan–Meier survival analysis was used to analyze the overall survival from the time of surgery. A log-rank test was used to compare the length of survival between curves. Statistical significance was set at a *P*-value of < 0.05. All statistical analyses were performed using SPSS version 19.0 (version 19.0. Armonk, NY: IBM Corp.).

### Fibroblast-driven ECM remodeling and SHG quantification using organotypic assay

Briefly, ∼7.5 × 10^4^/ml primary human fibroblasts were embedded in a three-dimensional matrix of rat tail collagen I. Rat tail tendon collagen solution was prepared by the extraction of tendons with 0.5 M acetic acid to a concentration of ∼0.5 mg/ml. Detached, polymerized matrix (2.5 ml) in 35-mm petri dishes was allowed to contract for approximately 8–12 days in complete media (DMEM, supplemented with 10% FCS, Invitrogen) + control, isotype control or LOX-blocking antibody (Timpson *et al*, 2011a,b). Using multiphoton imaging and a Ti:Sapphire femtosecond laser cavity (Coherent Chameleon Ultra II), coupled into a LaVision Biotec Trim-scope scan head, 890 nm excitation wavelength was used to collect SHG signal (435 ± 20 nm) from collagen from three separate matrices, within three different zones as previously described (Samuel *et al*, 2011). A z-section of 80 μm within the matrix was used at 2.5-μm intervals to collect a 3D representation of the matrix ultrastructure as previously described (Samuel *et al*, 2011). ImageJ was used to calculate ROI covered by SHG/fibrillar ECM signal per optical slice within the volume, after conversion to a binary image based upon threshold values. Columns, mean; error bars represent ± SE for respective treatment, *P* < 0.01 by unpaired Student’s *t*-test.

### Allograft mouse models

All experiments were performed in accordance with the local ethical review panel, the UK Home Office Animals Scientific Procedures Act, 1986 and UKCCCR and NCRI guidelines (Workman *et al*, 2010). Mouse tumor allografts were obtained using subcutaneous injection (s.c.) of 2 × 10^6^ cells in CD1 nude mice (Harlan). Tumors and organs were fixed in 10% buffered formalin overnight; fixed tissues were paraffin-embedded, and 5-μm sections were placed on sialylated/poly-l-lysine slides for further analysis. Tumor burden was assessed on the hematoxylin and eosin staining (H&E) of the lungs by two observers, inter-observer agreement quadratic weighted kappa = 0.79 (good agreement).

### Genetically modified mice and animal care

Animals were kept in conventional animal facilities and monitored daily, and all experiments were carried out in compliance with UK Home Office guidelines. Mice were genotyped by Transnetyx (Cordova, TN). Mice were treated with 1 mg/kg LOX-blocking antibody or isotype control antibody in PBS, and/or 100 mg/kg gemcitabine, twice weekly by i.p. injection. Tumor and metastatic burden was assessed by gross pathology and histology. Animals were sacrificed by cervical dislocation as per the institutional guidelines, and organs and tumors were removed and either fixed in 10% buffered formalin overnight at room temperature or snap-frozen in liquid nitrogen. Fixed tissues were paraffin-embedded, and 5-μm sections were placed on sialynated/poly-l-lysine slides for immunohistochemical analysis.

### Immunohistochemistry

Formalin-fixed paraffin-embedded sections were deparaffinized and rehydrated by passage through xylene and a graded alcohol series. Endogenous peroxidase activity was inactivated by treatment with 3% hydrogen peroxide, after which antigen retrieval was performed by incubation in citrate buffer in a pressure cooker. Sections were blocked in 5% serum for an hour and then incubated with primary antibody overnight at 4°C. Primary antibodies used were anti-CD31 (Abcam) 1:100, anti-MPO (Dako) 1:1,000, anti-F4/80 (Abcam) 1:100, anti-tenascin (Sigma) 1:2,000, anti-Ki-67 (Vector) 1:200 and anti-cleaved caspase-3 (R&D) 1:800. Sections were incubated in secondary antibody for 30 min (Vectastain ABC system) and staining visualized with 3,3′-diaminobenzidine tetrahydrochloride. Sirius red staining was carried out as described previously.

## Supporting Information

**Source Data for Supplementary Figure S2**

**Review Process File**
