# Supplementary figures and images for: Targeting the LOX/hypoxia axis reverses many of the features that make pancreatic cancer deadly: inhibition of LOX abrogates metastasis and enhances drug efficacy
Source: EMBO Mol Med. 2015 Jun 15;7(8):1063–76. doi: 10.15252/emmm.201404827 (PMC4551344; doi:10.15252/emmm.201404827)

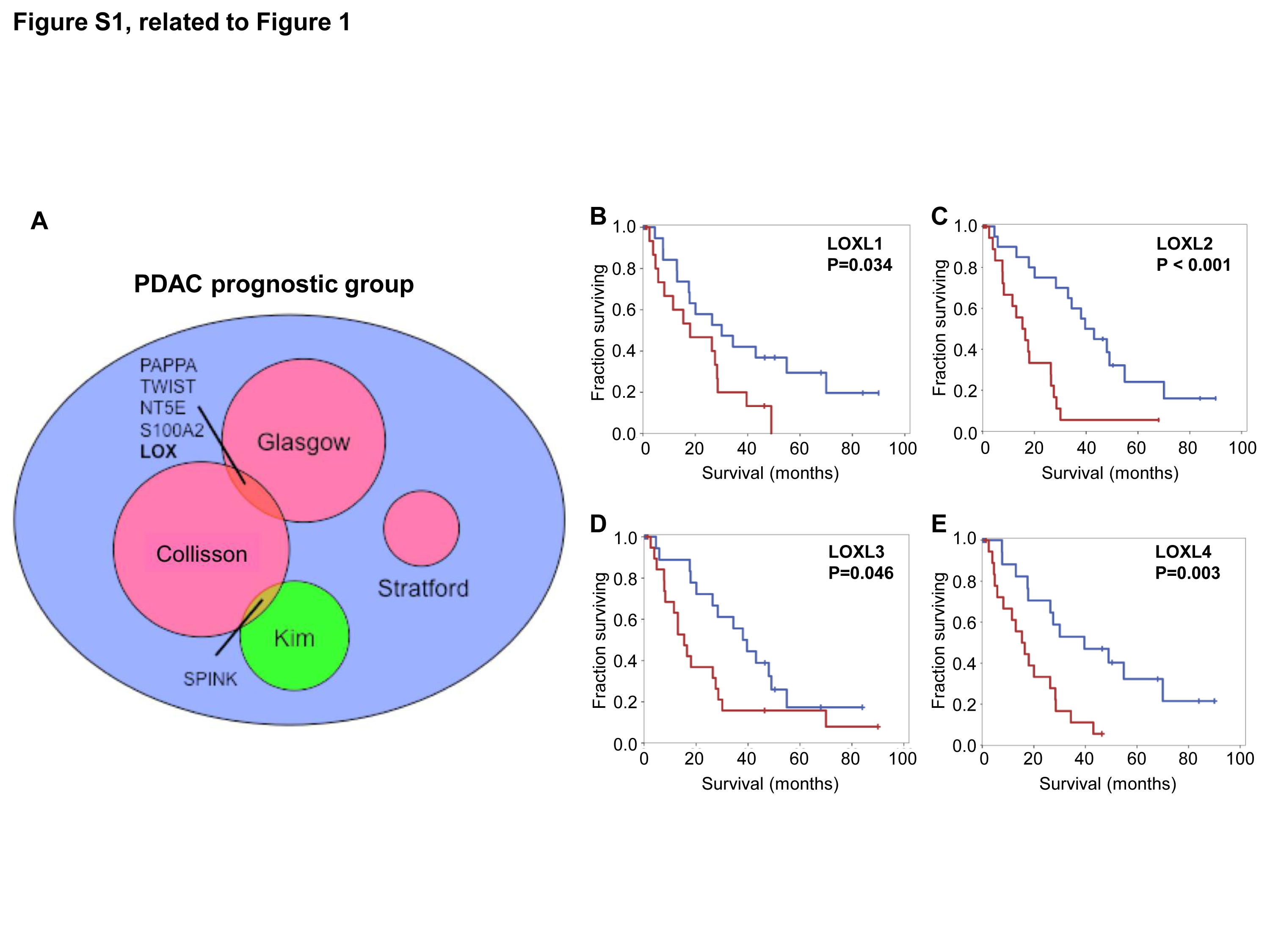

Supplement: Supplementary file 1 [file emmm0007-1063-sd1.tif]

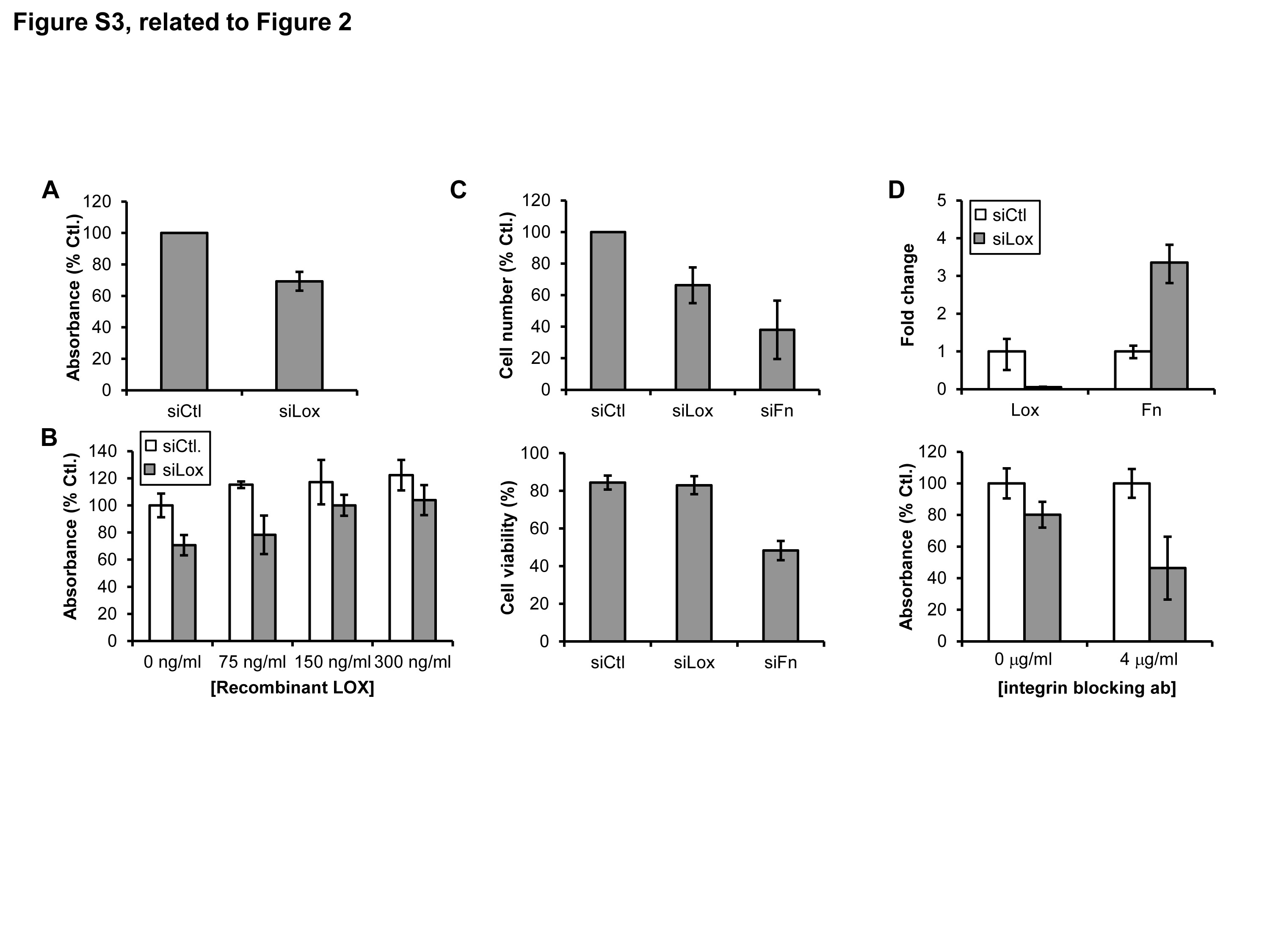

Supplement: Supplementary file 3 [file emmm0007-1063-sd3.tif]

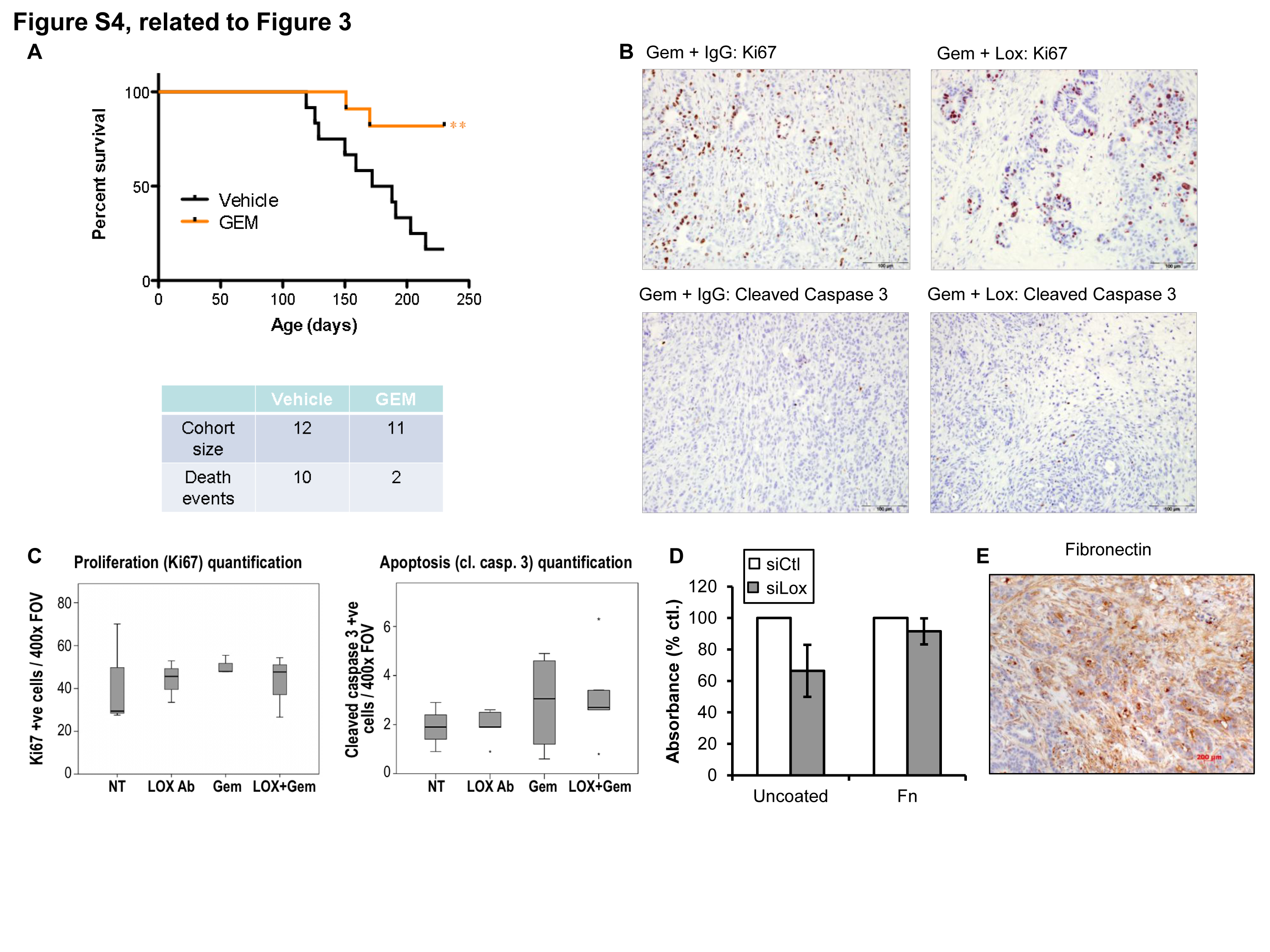

Supplement: Supplementary file 4 [file emmm0007-1063-sd4.tif]

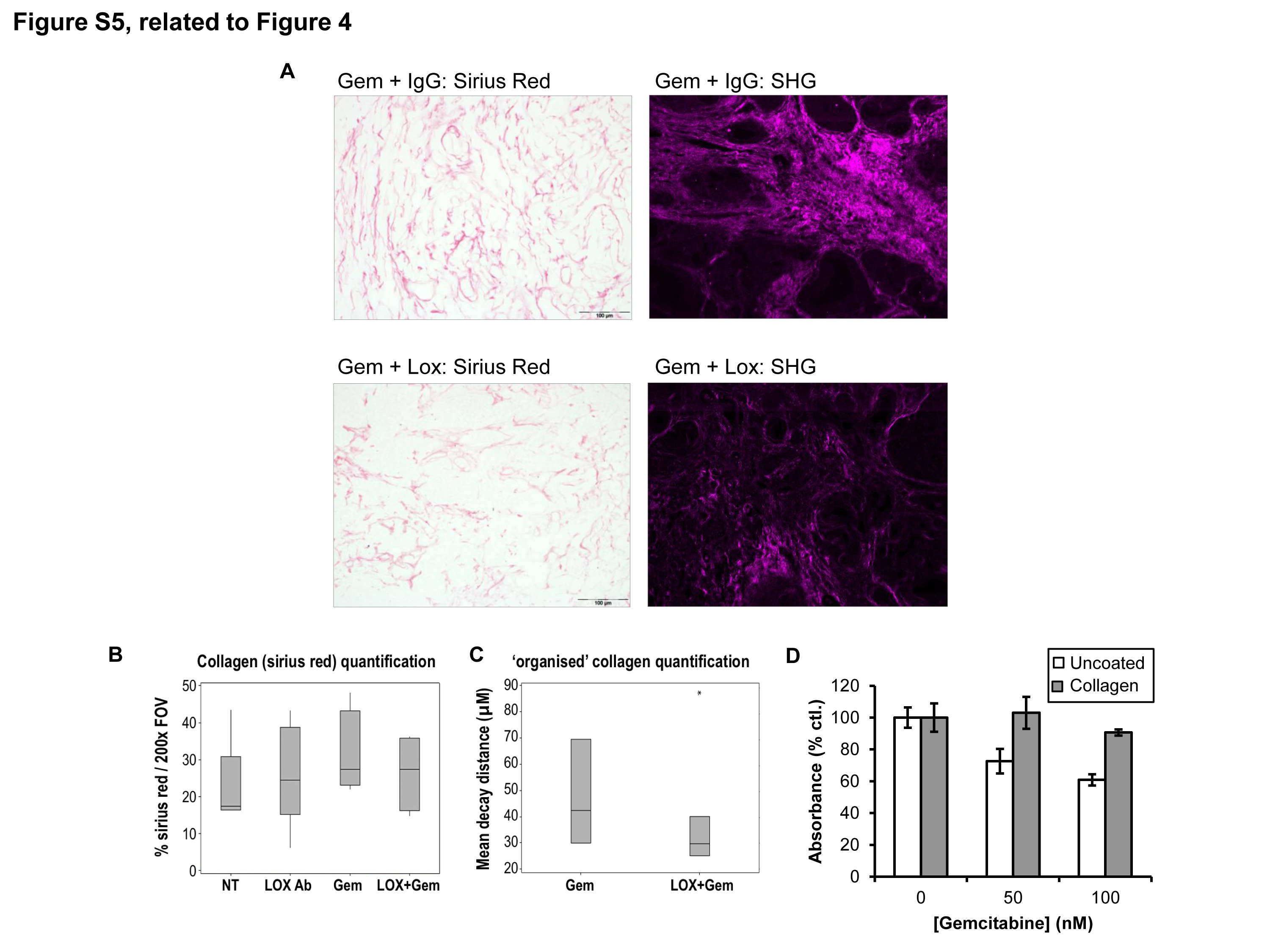

Supplement: Supplementary file 5 [file emmm0007-1063-sd5.tif]

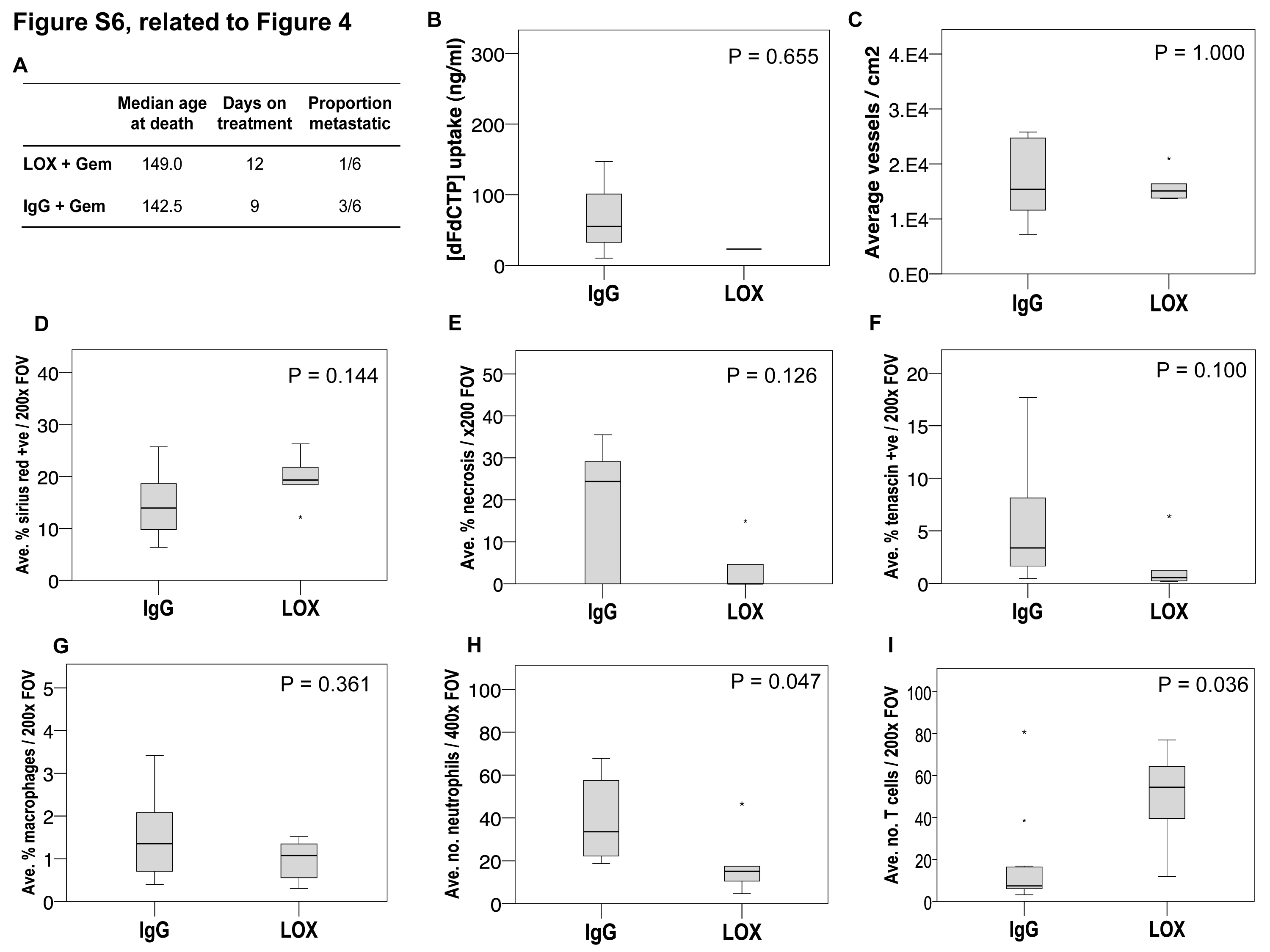

Supplement: Supplementary file 6 [file emmm0007-1063-sd6.tif]

**Supplementary Table S4: Quantitation of gemcitabine penetration**


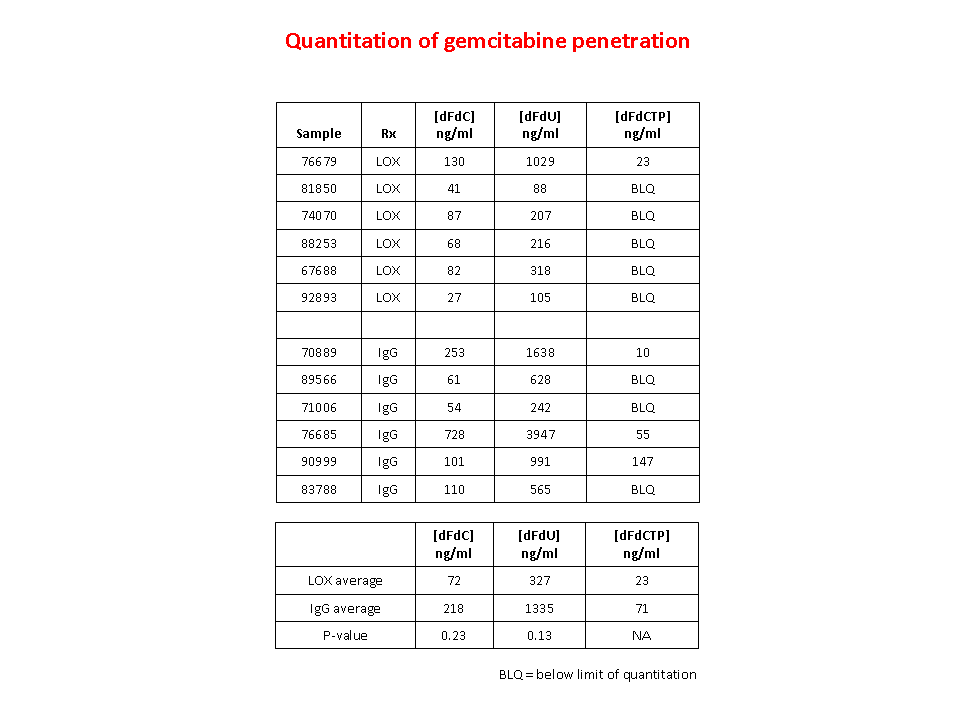

Supplement: Supplementary file 11 [file emmm0007-1063-sd11.docx]

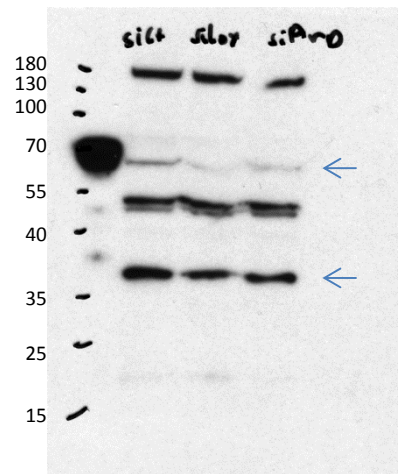

LOX (high exposure)

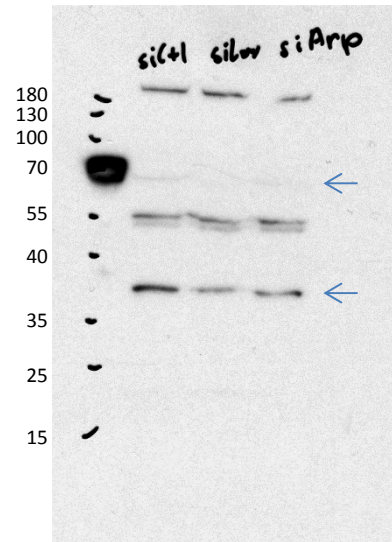

LOX (low exposure)

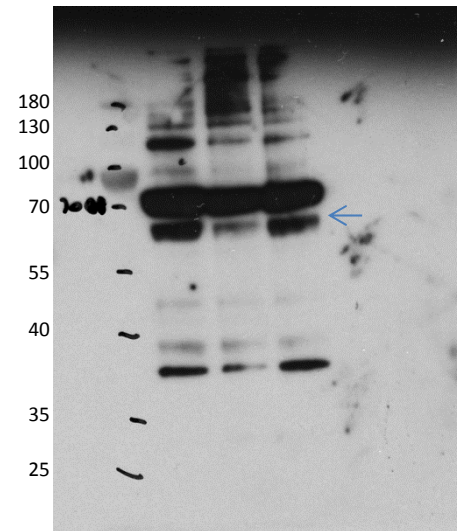

pSrc\_pYes

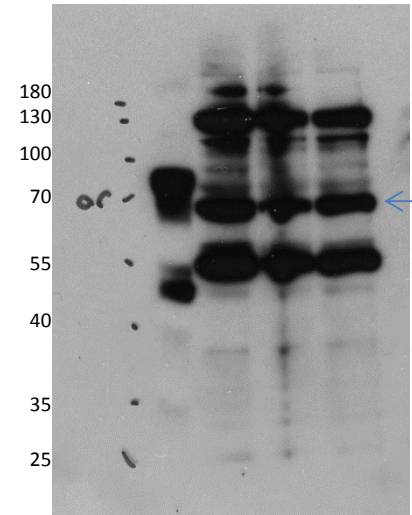

Total Src

Figure S2

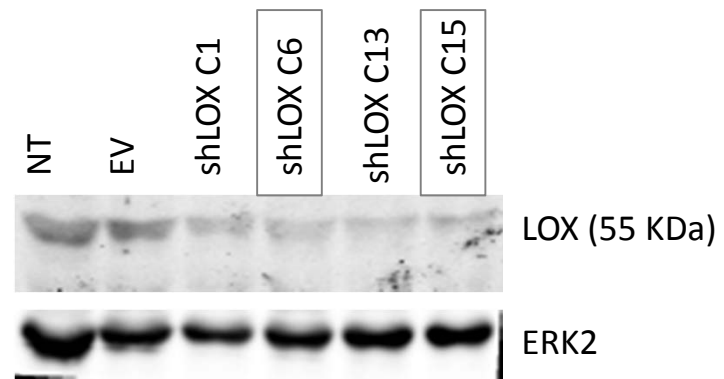

Figure S2

Supplement: Supplementary file 12 [file emmm0007-1063-sd12.pdf]

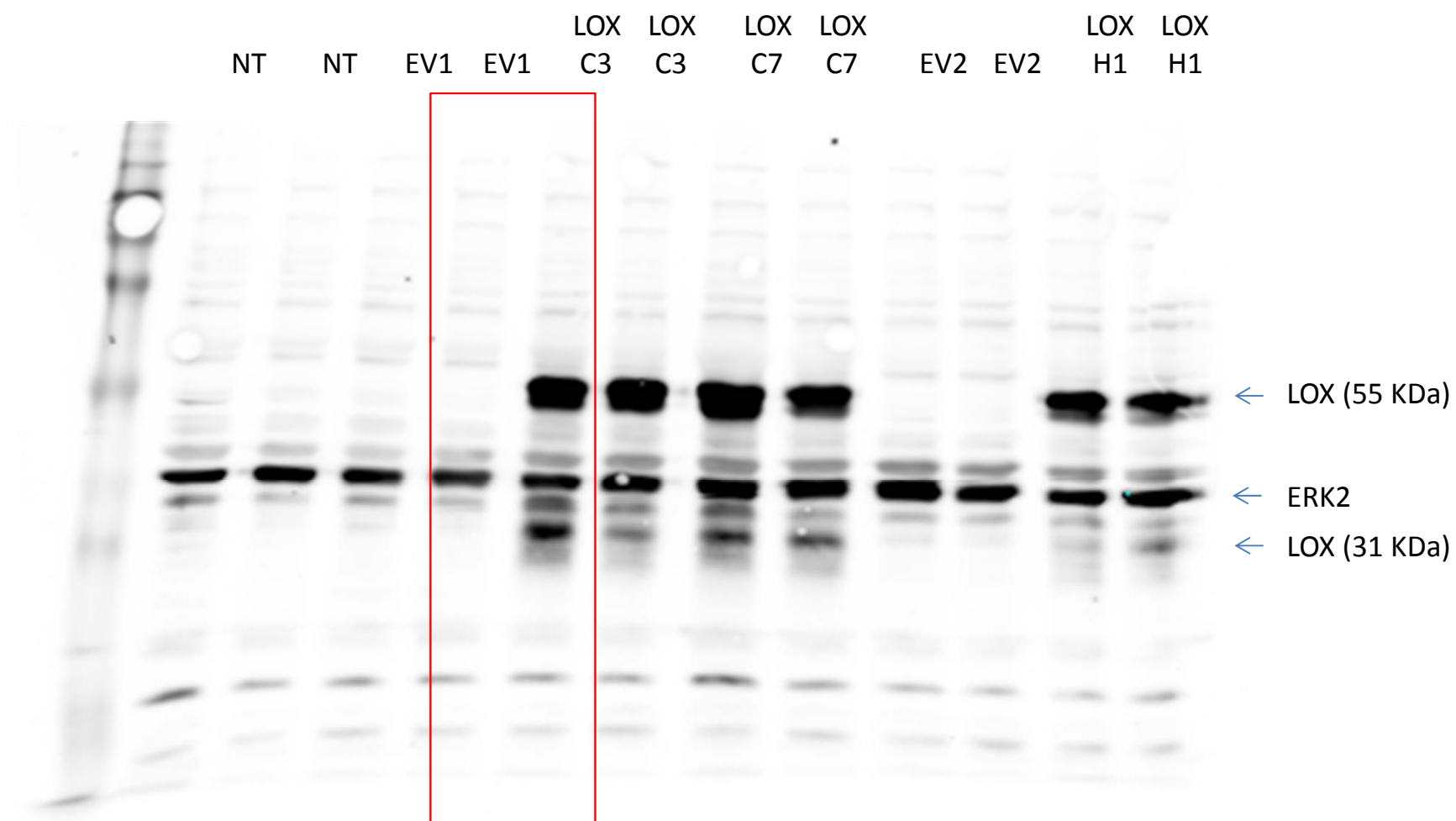

Figure 2

Supplement: Supplementary file 14 [file emmm0007-1063-sd14.pdf]
