# Supplementary material for: Targeting the LOX/hypoxia axis reverses many of the features that make pancreatic cancer deadly: inhibition of LOX abrogates metastasis and enhances drug efficacy
Source: EMBO Mol Med. 2015 Jun 15;7(8):1063–76. doi: 10.15252/emmm.201404827 (PMC4551344; doi:10.15252/emmm.201404827)
Supplement: Supplementary file 7 [file emmm0007-1063-sd7.docx]

**Supplementary Figure Legends**

**Supplementary Figure S1, related to Figure 1**

(A) Schematic showing that *LOX* is found in the overlap between the Glasgow cohort and the Collisson signature of poor prognosis in pancreatic cancer. The Stratford prognosis signature revealed no overlap while the Kim signature examined expression stratified by lymph node metastasis ([Collisson et al., 2011](#_ENREF_10); [Jamieson et al., 2011](#_ENREF_21); [Kim et al., 2012](#_ENREF_24); [Stratford et al., 2010](#_ENREF_39)). (B-E) Kaplan-Meier analyses showing correlation of LOX family member expression and survival in the Glasgow patient cohort. The upper quartile of LOX expressors are shown in red while the lower quartile is shown in blue.

**Supplementary Figure S2, related to Figure 2**

(A) LOX knockdown inhibits KPC cell migration. KPC cells were transfected with siRNA targeting LOX or with control siRNA, and culture media was supplemented with recombinant LOX protein (rLOX) where indicated. Migration of the cells was monitored using an Incucyte system. Quantitation is shown as the average relative wound density of four wells +/- standard deviation. (B) LOX knockdown in KPC cells is associated with a reduction in SRC phosphorylation. Cells were transfected with the indicated siRNAs and protein levels assessed by western blotting. (C) Dasatinib inhibits KPC cell migration. Quantitation is shown as the average relative wound density of three wells +/- standard deviation. (D) LOX knockdown inhibits migration in human PDAC cells. Panc1 cells were transfected with the indicated siRNAs and quantitation is shown as the average wound density of four wells +/- standard deviation. (E) Expression of LOX family members in KPC and KP^fl^C cells was compared by QPCR. Data are expressed as fold increase of expression in KPC cells relative to KP^fl^C and error bars show standard deviation. (F) Cells were either non-transfected (NT), transfected with empty vector (EV) or transfected with shRNA targeting LOX and protein levels were assessed by western blotting. (G) Growth curves of KPC cells grown as subcutaneous allografts on the flank of CD1-Nude mice show that LOX knockdown slows the growth of KPC tumors. Data are shown as average tumor volume (4 mice/group) +/- SEM. (H) Growth curves of KP^fl^C cells grown as subcutaneous allografts on the flank of CD1-Nude mice show that exogenous LOX enhances the growth of KP^fl^C tumors (8 mice/group left graph, 4 mice/group right graph). Data are shown as average tumor volume +/- SEM.

**Supplementary Figure S3, related to Figure 2**

(A) Knockdown of LOX causes a reduction in viable KPC cells. Transfection was carried out with the indicated siRNAs. Following 72 hours, cell viability was assessed using cell-titer blue. Data are shown as the mean of three independent experiments +/- SEM. (B) The reduction in viable cell number by Lox knockdown can be rescued by recombinant LOX protein. Following transfection, culture medium was supplemented with the indicated concentrations of LOX protein. Cell viability was measured using cell-titer blue and data are shown as the average of 3 wells +/- standard deviation. (C) KPC cells were transfected with the indicated siRNAs. Live and dead cell number was assessed by trypan blue exclusion. Data are expressed as the mean of three independent experiments +/- SEM. (D) *Top panel:* Expression of *Lox* and *Fn1* was detected by QPCR. Data are shown as the average of 3 wells +/- range. *Bottom panel:* KPC cells were transfected with the indicated siRNAs and incubated with or without mAb16 α5-integrin blocking antibody. Viable cell number was measured using cell-titer blue and data are expressed as the average of three wells +/- standard deviation.

**Supplementary Figure S4, related to Figure3**

(A) Gemcitabine treatment is chemopreventative. Mice were dosed with 100 mg/kg gemcitabine twice weekly commencing at 5-6 weeks of age. Treatment was ended at 32-33 weeks of age. Survival is shown as a Kaplan Meier plot. (B) *Upper panels*: Immunohistochemical staining for Ki67 in tumors from isotype control + gemcitabine treated KPC mice vs. LOX antibody + gemcitabine treated KPC mice. *Lower panels*: Immunohistochemical staining for cleaved caspase 3 in tumors from isotype control + gemcitabine treated KPC mice vs. LOX antibody + gemcitabine treated KPC mice. (C) Boxplots showing quantification of Ki67positive proliferating cells (left graph) and cleaved caspase 3 positive apoptotic cells (right graph) in tumors from KPC mice treated as indicated. At least 40 fields of view from 5 mice per cohort were scored. (D) KPC cells were grown on uncoated or fibronectin coated plates and transfected with the indicated siRNAs. Following 72 hours transfection, viable cell number was measured using cell-titer blue. Data are shown as the mean of three independent experiments +/- SEM. (E) Fibronectin was detected by immunohistochemistry in a KPC mouse tumor.

**Supplementary Figure S5, related to Figure 4**

(A) *Left panels*: Representative images of Sirius red staining for collagen in KPC mice treated with isotype control + gemcitabine vs. LOX antibody + gemcitabine. *Right panels*: Representative images of SHG signal in KPC mice treated with gemcitabine vs. LOX antibody + gemcitabine. (B) Boxplot of total collagen percentage per field of view in tumors from KPC mice treated as indicated. At least 30 fields of view from 5 mice per cohort were scored. (C) Boxplot of mean decay distances of the second harmonic generation (SHG) signal emitted by PDAC-associated collagen in tumors from KPC mice treated as indicated. At least 5 fields of view from 5 mice per cohort were scored. (D) KPC cells grown on uncoated or collagen coated plates were treated with the indicated concentration of gemcitabine for 24 hours. Cell viability was assessed using cell-titer blue and data are shown as the average of 3 wells +/- standard deviation.

**Supplementary Figure S6, related to Figure 4**

LOX inhibition, in combination with gemcitabine, is less effective in symptomatic KPC mice. (A) Table showing the effects of LOX + Gemcitabine treatment on survival and metastasis in symptomatic mice. (B-I) Boxplots showing quantification of: (B) intratumoral Gemcitabine metabolite dFdCTP; (C) tumor vasculature by CD31 IHC; (D) picrosirius red IHC; (E) necrosis; (F) tenascin C IHC; (G) macrophage infiltration by F480 IHC; (H) neutrophil infiltration by MPO IHC and (I) T cell infiltration by CD3 IHC, in tumors from KPC mice treated as indicated. At least 30 fields of view from 5 mice per cohort were scored. Mann-Whitney P values are indicated.
