# Supplementary material for: Targeting the LOX/hypoxia axis reverses many of the features that make pancreatic cancer deadly: inhibition of LOX abrogates metastasis and enhances drug efficacy
Source: EMBO Mol Med. 2015 Jun 15;7(8):1063–76. doi: 10.15252/emmm.201404827 (PMC4551344; doi:10.15252/emmm.201404827)
Supplement: Supplementary file 8 [file emmm0007-1063-sd8.docx]

**Supplementary Table S1, related to Figure 1**

| **Hazardous Component: 289 transcripts** | |
| --- | --- |
| **MSigDB Gene Set** | **FDR (BY)** |
| Winter_hypoxia_metagene | 0.000 |
| Elvidge_hypoxia_up | 0.000 |
| Bild_hras_oncogenic_signature | 0.000 |
| Jiang_hypoxia_normal | 0.000 |
| Charafe_breast_cancer_luminal_vs_basal_dn | 0.000 |
| Koinuma_targets_of_smad2_or_smad3 | 0.000 |
| Mense_hypoxia_up | 0.001 |
| Elvidge_hypoxia_by_dmog_up | 0.002 |
| Charafe_breast_cancer_luminal_vs_mesenchymal_dn | 0.002 |
| Senese_hdac1_targets_up | 0.002 |
| Buytaert_photodynamic_therapy_stress_up | 0.003 |
| Kan_response_to_arsenic_trioxide | 0.009 |
| Huang_dasatinib_resistance_up | 0.016 |
| Kobayashi_Egfr_Signaling_6hr_Dn | 0.024 |
| Mahadevan_response_to_mp470_dn | 0.024 |
| Wiederschain_targets_of_bmi1_and_pcgf2 | 0.024 |
| Plasari_Tgfb1_Targets_10hr_Up | 0.024 |
| Manalo_hypoxia_up | 0.025 |
| Zwang_class_1_transiently_induced_by_egf | 0.025 |
| Gross_hypoxia_via_elk3_dn | 0.031 |
| Davicioni_targets_of_pax_foxo1_fusions_up | 0.035 |
| Nuytten_ezh2_targets_up | 0.036 |
| Podar_response_to_adaphostin_up | 0.036 |
| Wang_methylated_in_breast_cancer | 0.036 |
| Rickman_tumor_differentiated_well_vs_poorly_dn | 0.041 |
| Gross_hypoxia_via_elk3_and_hif1a_up | 0.044 |
| Lien_breast_carcinoma_metaplastic_vs_ductal_up | 0.048 |
| **Protective component: 253 transcripts** | |
| **MSigDB Gene Set** | **FDR (BY)** |
| Smid_breast_cancer_normal_like_up | 0.000 |
| Wallace_prostate_cancer_race_up | 0.001 |
| Lee_differentiating_t_lymphocyte | 0.003 |
| Chr1q32 | 0.009 |
| Klein_primary_effusion_lymphoma_dn | 0.016 |
| Shedden_lung_cancer_good_survival_a12 | 0.031 |
| Kegg_antigen_processing_and_presentation | 0.036 |
| Pasqualucci_lymphoma_by_gc_stage_dn | 0.048 |
| Yu_myc_targets_dn | 0.050 |
| Smid_breast_cancer_luminal_b_dn | 0.050 |
