# Supplementary material for: Targeting the LOX/hypoxia axis reverses many of the features that make pancreatic cancer deadly: inhibition of LOX abrogates metastasis and enhances drug efficacy
Source: EMBO Mol Med. 2015 Jun 15;7(8):1063–76. doi: 10.15252/emmm.201404827 (PMC4551344; doi:10.15252/emmm.201404827)
Supplement: Supplementary file 9 [file emmm0007-1063-sd9.docx]

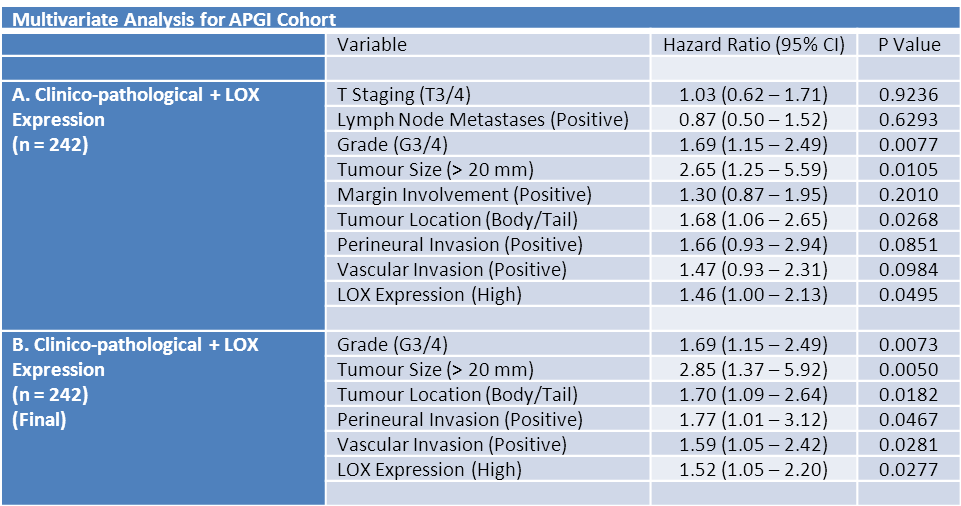


**Supplementary Table S2: Multivariate analysis**

**A:** Starting model of multivariate analysis

**B:** Final resolved model (all variables independently prognostic)
