# Supplementary material for: Targeting the LOX/hypoxia axis reverses many of the features that make pancreatic cancer deadly: inhibition of LOX abrogates metastasis and enhances drug efficacy
Source: EMBO Mol Med. 2015 Jun 15;7(8):1063–76. doi: 10.15252/emmm.201404827 (PMC4551344; doi:10.15252/emmm.201404827)
Supplement: Supplementary file 13 [file emmm0007-1063-sd13.pdf]

Targeting the LOX/HYPOXIA axis reverses many of the features that make pancreatic cancer deadly: Inhibition of LOX abrogates metastasis and enhances drug efficacy

Bryan W. Miller, Jennifer P. Morton, Mark Pinese, Grazia Saturno, Nigel B. Jamieson, Ewan McGhee, Paul Timpson, Joshua Leach, Lynn McGarry, Emma Shanks, Peter Bailey, David Chang, Karin Oien, Saadia Karim, Amy Au, Colin Steele, C. Ross Carter, Colin McKay, Kurt Anderson, T.R. Jeffry Evans, Richard Marais, Caroline Springer, Andrew Biankin, Janine Erler, Owen J Sansom

Corresponding author: Owen Sansom, Cancer Research UK Beatson Laboratories

Review timeline:

Submission date:	09 November 2014
Editorial Decision:	18 December 2014
Revision received:	30 March 2015
Editorial Decision:	28 April 2015
Revision received:	12 May 2015
Accepted:	21 May 2015

Transaction Report:

(Note: With the exception of the correction of typographical or spelling errors that could be a source of ambiguity, letters and reports are not edited. The original formatting of letters and referee reports may not be reflected in this compilation.)

Editor: Roberto Buccione

1st Editorial Decision 18 December 2014

We have now received comments from the two out of the three Reviewers whom we asked to evaluate your manuscript. As I had mentioned, we are however experiencing significant delay in obtaining the third from Reviewer 3.

As a further delay cannot be justified, I have decided to proceed based on the available evaluations from Reviewers 1 and 2.

If in the meanwhile should we receive the third evaluation, but only if it raises significant caveats, these would need to be taken into consideration. We will not, however, ask you to comply with any further-reaching requests.

While Reviewer 1 is clearly positive, Reviewer 2 is more reserved. In aggregate, they raise a number of issues that require your action. I will not go into little detail as their comments are quite clear and I will therefore simply highlight the main ones.

Reviewer 1 would like you to document LOX expression and collagen features at later stages to solidify the clinical relevance of your observations. S/he would also like you to provide better, more conclusive data on the effects of LOX knockdown and to extend the invasion assays to the PDAC

human cell lines. A few other items are listed that require your attention.

Reviewer 2 feels that you should have presented data from larger cohorts and also that several important items are missing from the Kaplan-Meier analysis, which ultimately impairs conclusiveness and significance. S/he also wonders whether LOX levels, the hypoxia signature and collagen cross-linking might actually be epiphenomena and suggests that improved data should be provided to solve this issue. This Reviewer, as does Reviewer 1, would like to see more convincing invasion data. Concerns are also expressed about the lack of a full analysis on the type of infiltrating immune cells. Reviewer 2 lists a few other items of concern. I should mention that this Reviewer is quite adamant that you have indulged in excessive "self-citation". I would suggest that you improve the manuscript in this respect, especially concerning the discussion of opposing views.

While publication of the paper cannot be considered at this stage, we would be pleased to consider a revised submission, with the understanding that the Reviewers' concerns must be addressed including with additional experimental data where appropriate and that acceptance of the manuscript will entail a second round of review.

EMBO Molecular Medicine now requires a complete author checklist (http://embomolmed.embopress.org/authorguide#editorial3) to be submitted with all revised manuscripts. Provision of the author checklist is mandatory at revision stage; The checklist is designed to enhance and standardize reporting of key information in research papers and to support reanalysis and repetition of experiments by the community. The list covers key information for figure panels and captions and focuses on statistics, the reporting of reagents, animal models and human subject-derived data, as well as guidance to optimise data accessibility. I am attaching a copy of the checklist to this letter for your convenience, but should you have problems opening it, please refer to the link provided.

Please also note that it is EMBO Molecular Medicine policy to allow a single round of revision only and that, therefore, acceptance or rejection of the manuscript will depend on the completeness of your responses included in the next, final version of the manuscript.

As you know, EMBO Molecular Medicine has a "scooping protection" policy, whereby similar findings that are published by others during review or revision are not a criterion for rejection. However, I do ask you to get in touch with us after three months if you have not completed your revision, to update us on the status. Please also contact us as soon as possible if similar work is published elsewhere.

I look forward to seeing a revised form of your manuscript as soon as possible.

In the meanwhile, I wish you a great holiday season and a happy, successful 2015!

***** Reviewer's comments *****

Referee #1 (Comments on Novelty/Model System):

This is an interesting study with high translational value. It provides strong clinical and preclinical evidence supporting the targeting of lysyl oxidase as new avenue to develop therapeutic approaches for pancreatic cancer, a disease in great need for new treatment regimens. The models and methodologies are state-of-the-art. There are only few medium/minor weaknesses in the study (see below).

Referee #1 (Remarks):

Specific Comments:

1) Although the data supporting the suitability of LOX as a therapeutic target, the rationale of focusing on this molecule is not clear. Further description of the gene expression studies should be included to help define this rationale.

2) With the emerging role of the stroma as protective compartment in pancreatic cancer, the authors

should examine if at later stages (e.g. non- resectable stages) the expression of LOX and collagen cross-link levels are different compared to early states and if they have any correlation with clinical features of the disease.

3) The levels of collagen cross-link should be examined in the GEMM model at different stages.

4) The section of the expression of LOX in mice could be shorter and incorporated into the first section of the results. It will help the flow of the text.

5) The invasion assays should be repeated in PDAC human cell lines. Also, the effect of LOX knockdown on cell growth, viability, anoikis and differentiation (e.g. EMT) should be evaluated in the presence or absence of matrigel.

6) Is the antibody used in the in vivo experiments blocking all LOXs? If so individual knockdown should be done to established the contribution of each of these enzymes to the migration of PDAC cells?

7) The molecular mechanism underlying the pro-migratorial effect of LOX should be defined? Is it through the regulation of SRC-FAK signaling? Integrins?

8) The tumors from the models used in Supp Fig 2D-F and Supp Fig3A-B do not resemble the humans counterpart or the GEMM ones. They lack the typical desmoplastic reaction of pancreatic tumors. Then, the anti-tumoral effect seen in these mice is suggesting that the LOX would have a different mechanism of action? Control for the specificity of BAPN should be included in the experiment? Also, the levels of collagen cross-link should be examined in this model before and after treatment.

Referee #2 (Comments on Novelty/Model System):

The model systems are state-of-the-art, however all data are not presented, precluding thorough evaluation. The potential to impact clinically is in 5-10 years and therefore is of relevance. There are a number of groups working on this aspect. The referencing is inadequate: there is preponderance to self-citation ignoring other work. Balanced view is not achieved.

Referee #2 (Remarks):

Miller et al produce data to convince readers that LOX is an important aspect in targeting stroma of pancreatic cancer. A large amount of missing data precludes thorough review of this potentially exciting information. Suggested areas of improvement to allow a proper review either at EMBO Mol Med or another journal would be:
1. A balanced reference list: there is pre-ponderance to self-citation; which ignores other evidence, some of which may be contrary to author's current and previous findings. This relates to LOX in cancer and in particular pancreatic cancer (e.g., Wiel Cell Death Diff 2013), effect of p53 in PDAC progression, effect of immune infiltrate, use of model systems amongst others. Whilst acknowledging it is impossible to cite all work, contrary views should be juxta-posed in Introduction and Discussion.
2. The Kaplan-Meier survival graphs are from very small cohorts of patients. Both Glasgow and Australian cohorts are well-published. They are large data-sets. It would be preferred that the analysis was done on whole data sets rather than this piecemeal presentation. Numbers at risk are missing below each graph. Multivariable analysis is missing to ascertain whether LOX or hypoxia signatures were truly prognostic or type I error. This is particularly striking since the curves separate early: within 90 days and remain separated for same amount. Peri-operative mortality, rather than tumor recurrence would account for this early separation, since most good units would not have peri-operative mortality before 45 days. Peri-operative mortality may have no bearing LOX or hypoxia signature. Authors have access to larger data sets and larger cohorts which should be presented.
3. Data on transcriptome of 73 patients is not presented. There is no link to another publication or

depositing raw data on GEO or other repositories. In absence of this data (SF1 and ST1 are perfunctory), one cannot ascertain the true value of this experiment and its analysis. The logic of focusing in hypoxia signature cannot be fully evaluated.
4. If collagen cross-linking was significantly associate with tumor stage, lymph node invasion and vascular invasion, then these certainly suggest that the most important prognostic features are confunders for survival analysis. Thus LOX, hypoxia signature or Collagen cross-linking may be epiphenomenon driving prognosis. Convincing data need to be presented.
5. Invasion was carried through Matrigel matrix (SF2)? Very low Collagen content. So in vitro data do not measure collagen degradation.
6. Role of BAPN on other enzymes is not evaluated.
7. If LOX kd resulted in less viable cells (SF3) then less invasion may be a function of survival rather than reduced invasive ability.
8. Timing of experiments in KPC mice and KP[fl]C mice is not fully explained. As evident from Rhim and Olive papers on Shh signaling this has important bearing on interpretation.
9. Immune cell infiltrate focuses in macrophages and neutrophils, however most other data point to CD8 and MDSC being important. What was the role of these immune cells. Macrophages and neutrophils infiltrate as analysed by authors has shortcomings in terms of false positive staining of a number of other cell types in tissues.
10. Why was Tenascin C evaluated when previous data were on Collagen 1 and Fn.
11. The absence of metastasis in Lox Ab is not fully evaluated or explained. Why did these mice succumb?

1st Revision - authors' response 30 March 2015

Referee #1 (Comments on Novelty/Model System):

This is an interesting study with high translational value. It provides strong clinical and preclinical evidence supporting the targeting of lysyl oxidase as new avenue to develop therapeutic approaches for pancreatic cancer, a disease in great need for new treatment regimens. The models and methodologies are state-of-the-art. There are only few medium/minor weaknesses in the study (see below).

Referee #1 (Remarks):

Specific Comments:

1) Although the data supporting the suitability of LOX as a therapeutic target, the rationale of focusing on this molecule is not clear. Further description of the gene expression studies should be included to help define this rationale.

We focused on LOX as a target because in addition to our PC-1 signature analysis, it was also a hit in the Glasgow and Collisson data sets (Fig S1A). It was one of the few genes common to the multiple data sets, and this suggested its potential as a therapeutic target.

2) With the emerging role of the stroma as protective compartment in pancreatic cancer, the authors should examine if at later stages (e.g. non- resectable stages) the expression of LOX and collagen cross-link levels are different compared to early states and if they have any correlation with clinical features of the disease.

This is an interesting point. However given patients with non-resectable disease succumb very quickly and there is no clinical reason for biopsy of the primary tumour we were unable to get tissue from non-resectable individuals. We would highlight our data that the patients that have the highest LOX and collagen crosslinking still succumb very quickly to disease compared to patients with lower levels of these markers. It should also be noted (see point 3), that in our models when we treat mice with late stage disease with already high levels of collagen crosslinking LOX inhibition, the

© EMBO

inhibition of LOX does not reverse this which is why would suggest LOX therapy for patients with surgically resectable disease.

3) The levels of collagen cross-link should be examined in the GEMM model at different stages.

Pre-malignant PANIN lesions from the KPC mice have very low levels of collagen so there is little collagen crosslinking at this stage.

To better assess the impact of LOX inhibition on collagen crosslinking at late stage disease (when mice have an established large primary tumour), we assessed whether LOX blockade could reduce the collagen crosslinking and found that it was unable to. This fits well with the data in figure S6 that LOX inhibition did not improve survival in these mice (in contrast to when mice were treated at day 70). This is expected as collagen is covalently crosslinked by LOX so inhibition would only be able to prevent new collagen crosslinking.

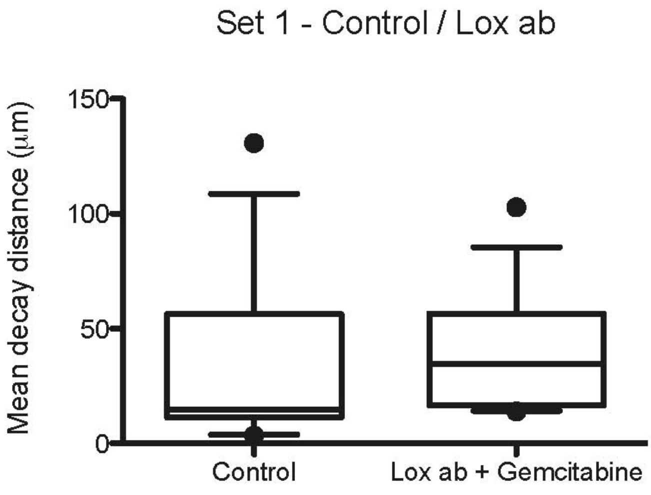

Mann Whitney test	
P value	0.1366
Exact or approximate P value?	Gaussian Approximation
P value summary	ns
Are medians signif. different? (P < 0.05)	No
One- or two-tailed P value?	Two-tailed
Sum of ranks in column B,C	224 , 304
Mann-Whitney U	88.00

These data suggest that the state of collagen cross linking in early and late stage disease is different and while anti-LOX therapy is effective when applied at stages prior to extensive cross-linking, it is less effective when applied to tumours with a high level of pre-existing cross-linked collagen.

4) The section of the expression of LOX in mice could be shorter and incorporated into the first section of the results. It will help the flow of the text.

We have reworked the text in this section and feel it improves the flow of the paper.

5) The invasion assays should be repeated in PDAC human cell lines. Also, the effect of LOX knockdown on cell growth, viability, anoikis and differentiation (e.g. EMT) should be evaluated in the presence or absence of matrigel.

We have reproduced the effect of LOX knockdown on wound healing in Panc-1 cells and have included this in the manuscript (Figure S2D).

We have also further investigated the means by which LOX knockdown results in fewer viable KPC cells. Using trypan blue exclusion, we quantitated the changes in live and dead cell number in response to LOX knockdown. We found that while LOX knockdown decreased live cell number, there was no corresponding increase in dead cells, suggesting that the effect we observed was due to decreased cell growth rather than decreased survival. These data have been included in the manuscript (Figure S3C).

To further investigate whether LOX was required for cell survival, we quantified anoikis in response to LOX knockdown. Consistent with the trypan data, we saw no increase in anoikis when LOX was targeted, even though total cell number was decreased.

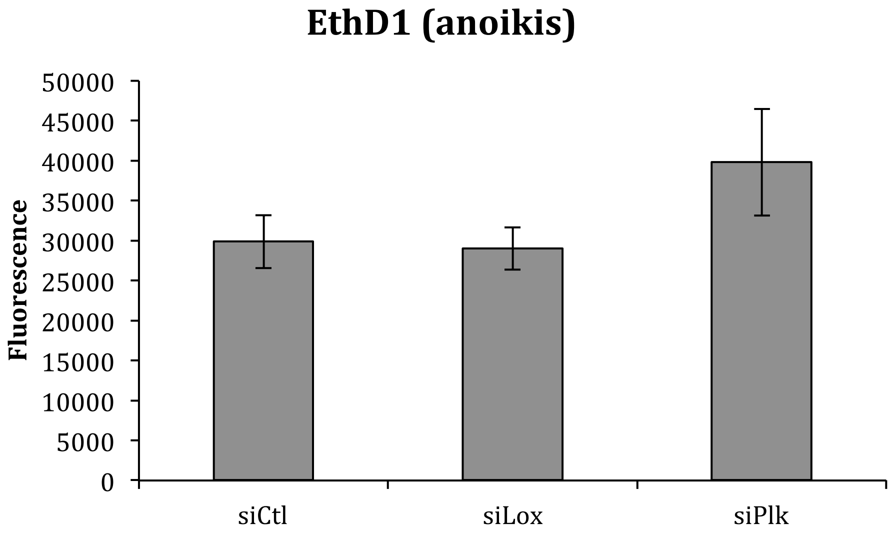

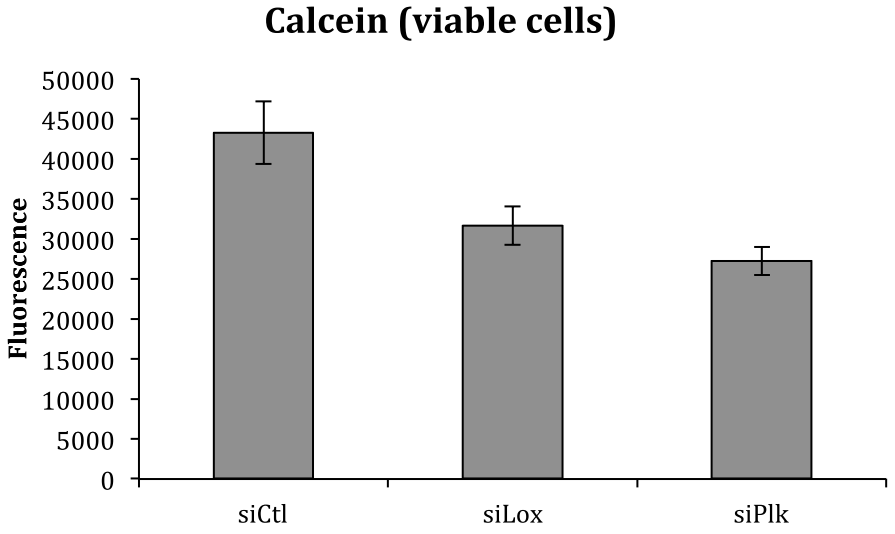

We also quantitated the expression of EMT markers in response to LOX knockdown. While we found a modest decrease in E-cadherin expression, we saw no change in expression of SNAIL or SLUG, suggesting that there was no significant effect on EMT when LOX was targeted.

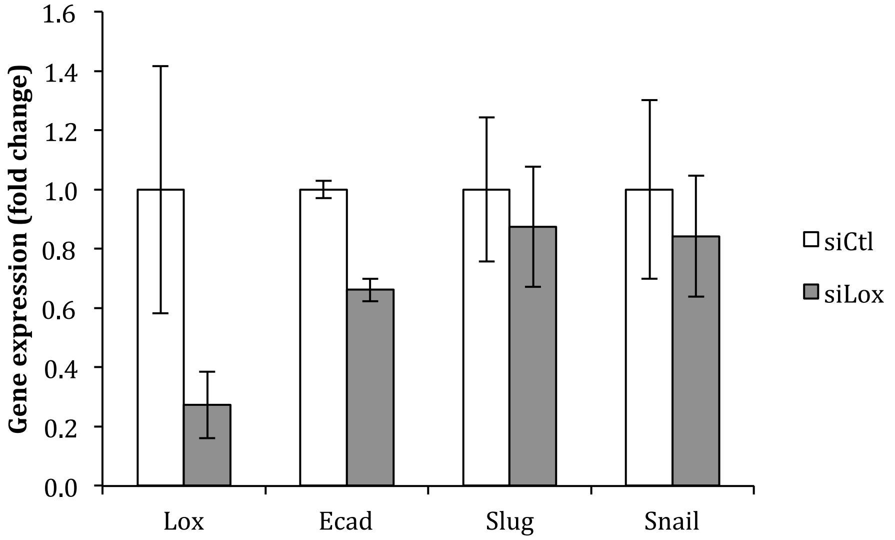

© EMBO

We also confirmed that the effects of LOX knockdown on reducing viable cell number were equivalent whether the cells were grown on matrigel or uncoated plates.

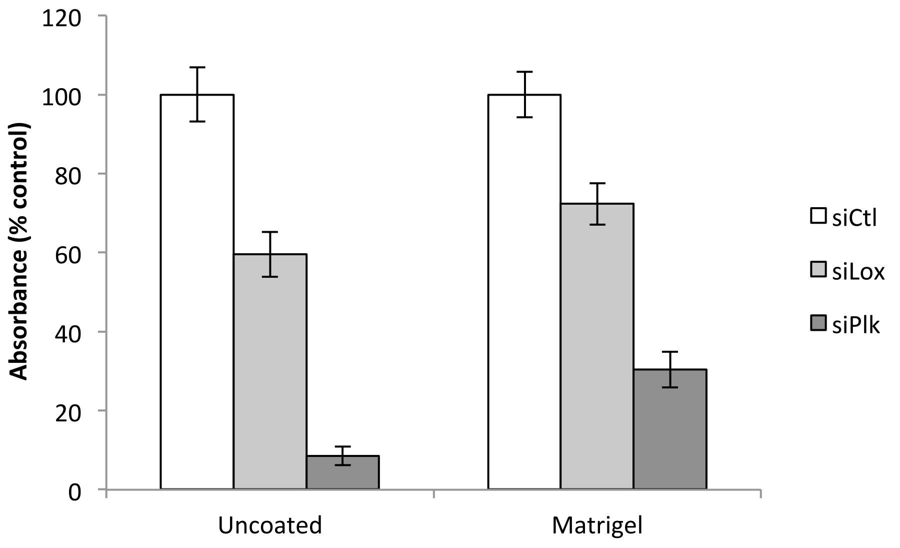

6) Is the antibody used in the in vivo experiments blocking all LOXs? If so individual knockdown should be done to established the contribution of each of these enzymes to the migration of PDAC cells?

The blocking antibody used is specific for LOX and does not recognise other family members. We have added text to the manuscript to clarify this.

7) The molecular mechanism underlying the pro-migratorial effect of LOX should be defined? Is it through the regulation of SRC-FAK signaling? Integrins?

This was excellent suggestion. We now provide evidence that effects of LOX on migration may in part be mediated by Src activation (Figure S2B-C). We consistently see loss of Src phosphorylation in these experiments when LOX is targeted and we can recapitulate the inhibition of migration using sub-lethal doses of a Src inhibitor. We have added data to the manuscript to this effect.

8) The tumors from the models used in Supp Fig 2D-F and Supp Fig3A-B do not resemble the humans counterpart or the GEMM ones. They lack the typical desmoplastic reaction of pancreatic tumors. Then, the anti-tumoral effect seen in these mice is suggesting that the LOX would have a different mechanism of action? Control for the specificity of BAPN should be included in the experiment? Also, the levels of collagen cross-link should be examined in this model before and after treatment.

We have decided to remove the BAPN data from the manuscript as it does inhibit multiple members of the LOX family and we want to focus on LOX as a therapeutic target.

We show elsewhere in the manuscript that targeting LOX can have direct effects on tumour cells in certain contexts and can slow cell growth in an ECM-dependent manner (figure S3 and S4). We hypothesised that due to the fact that the subcutaneous tumours will not recapitulate the stromal conditions of a mature PDAC, the effects of modulating LOX expression will be due to effects on the tumour cells directly.

In order to test this, we addressed whether altering LOX expression altered levels of collagen organisation in the subcutaneous tumours. We saw no evidence of this, suggesting that modulation of LOX primarily acted by slowing tumour cell growth.

© EMBO

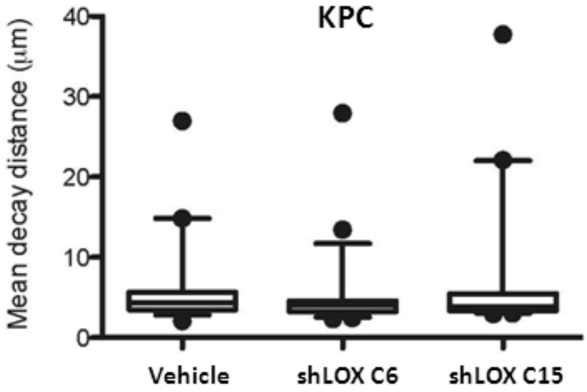

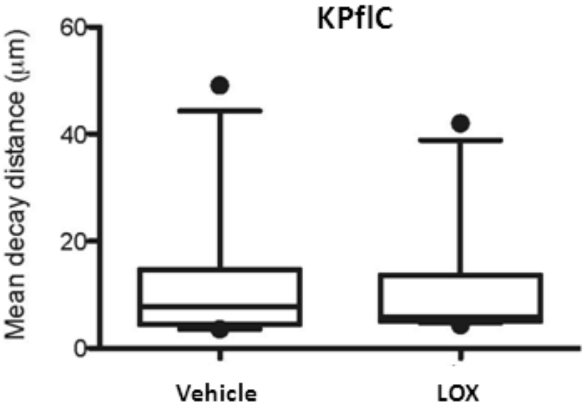

Referee #2 (Comments on Novelty/Model System):

The model systems are state-of-the-art, however all data are not presented, precluding thorough evaluation. The potential to impact clinically is in 5-10 years and therefore is of relevance. There are a number of groups working on this aspect. The referencing is inadequate: there is preponderance to self-citation ignoring other work. Balanced view is not achieved.

Referee #2 (Remarks):

Miller et al produce data to convince readers that LOX is an important aspect in targeting stroma of pancreatic cancer. A large amount of missing data precludes thorough review of this potentially exciting information. Suggested areas of improvement to allow a proper review either at EMBO Mol Med or another journal would be:

1. A balanced reference list: there is pre-ponderance to self-citation; which ignores other evidence, some of which may be contrary to author's current and previous findings. This relates to LOX in cancer and in particular pancreatic cancer (e.g., Wiel Cell Death Diff 2013), effect of p53 in PDAC progression, effect of immune infiltrate, use of model systems amongst others. Whilst acknowledging it is impossible to cite all work, contrary views should be juxta-posed in Introduction and Discussion.

We apologise for missing a number of citations in the previous version and have added a more comprehensive overview of the previous literature.

2. The Kaplan-Meier survival graphs are from very small cohorts of patients. Both Glasgow and Australian cohorts are well-published. They are large data-sets. It would be preferred that the analysis was done on whole data sets rather than this piecemeal presentation. Numbers at risk are missing below each graph. Multivariable analysis is missing to ascertain whether LOX or hypoxia signatures were truly prognostic or type I error. This is particularly striking since the curves separate early: within 90 days and remain separated for same amount. Peri-operative mortality, rather than tumor recurrence would account for this early separation, since most good units would not have peri-operative mortality before 45 days. Peri-operative mortality may have no bearing LOX or hypoxia signature. Authors have access to larger data sets and larger cohorts which should be presented.

As the reviewer suggested, we have repeated the analysis on the larger APGI cohort consisting of 266 patients and also included multivariate analysis. The results were consistent with our previous work and showed a strong correlation between LOX expression and patient survival. These data have been incorporated into the paper along with the additional information requested.

3. Data on transcriptome of 73 patients is not presented. There is no link to another publication or depositing raw data on GEO or other repositories. In absence of this data (SF1 and ST1 are perfunctory), one cannot ascertain the true value of this experiment and its analysis. The logic of focusing in hypoxia signature cannot be fully evaluated.

The gene expression data have been deposited in GEO and we have added this information to the manuscript. http://www.ncbi.nlm.nih.gov/geo/query/acc.cgi?acc=GSE50827

4. If collagen cross-linking was significantly associated with tumor stage, lymph node invasion and vascular invasion, then these certainly suggest that the most important prognostic features are confunders for survival analysis. Thus LOX, hypoxia signature or Collagen cross-linking may be epiphenomenon driving prognosis. Convincing data need to be presented.

We now have provided a multivariate analysis (Table S2). LOX was able to predict prognosis independently of any others factor.

5. Invasion was carried through Matrigel matrix (SF2)? Very low Collagen content. So in vitro data do not measure collagen degradation.

While the initial transient experiments did use matrigel, the invasion panels in the main body of the paper (Figure2B-D) were carried out using collagen.

6. Role of BAPN on other enzymes is not evaluated.

In light of the fact that the paper is focused on the specific targeting of LOX with the blocking antibody, we decided to remove the BAPN data to ensure a clearer focus on the targeting of LOX alone.

7. If LOX kd resulted in less viable cells (SF3) then less invasion may be a function of survival rather than reduced invasive ability.

The difference we see in cell numbers is due to a difference in cell growth rather than survival, and we have added these data to the manuscript (Figure S3C). While this result is highly reproducible at lower cell densities, at the higher densities used for the invasion assays no difference in cell number is observed.

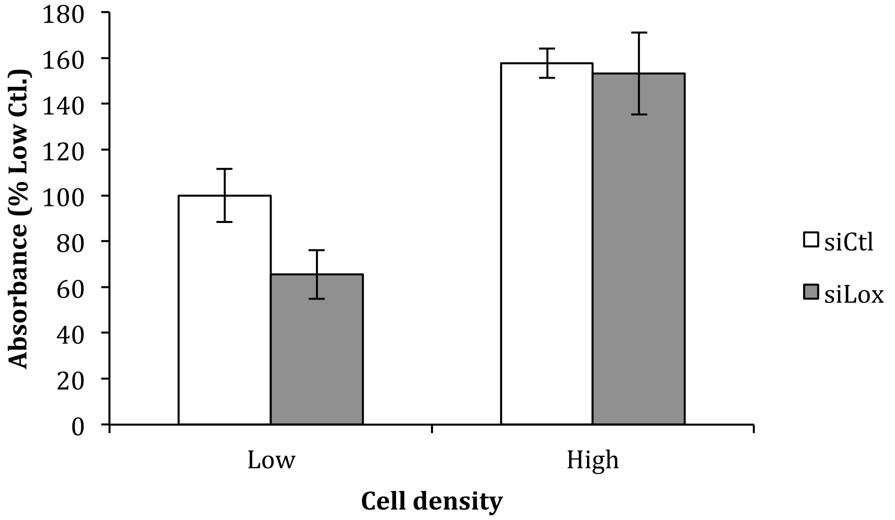

In order to confirm this, we repeated the invasion assay and incorporated a resazurin assay to quantitate viable cells. This confirmed that while we saw a slowing of invasion in response to LOX knockdown, there was no effect on overall cell number.

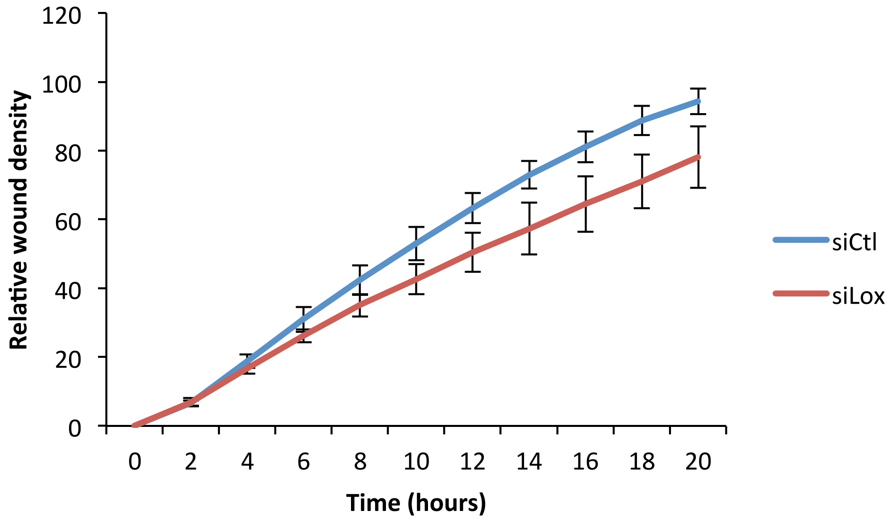

© EMBO

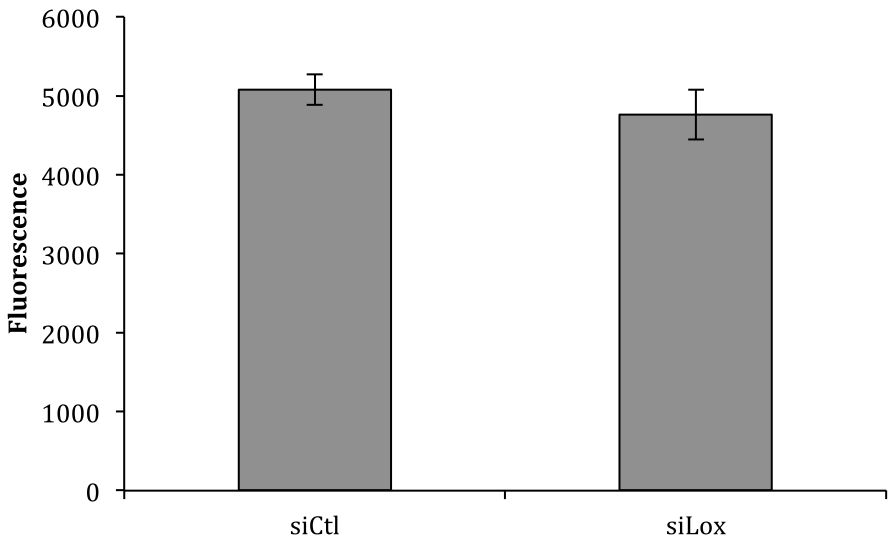

8. Timing of experiments in KPC mice and KP[fl]C mice is not fully explained. As evident from Rhim and Olive papers on Shh signaling this has important bearing on interpretation.

We agree that the time point is important and have added text to the manuscript explaining our rationale for selecting the 70 day time point to commence treatment. Importantly we have performed both an early timepoint (equivalent to the Rhimm paper where they see acceleration of tumourigenesis with hedgehog inhibition) and late stage (where Olive saw a slowing of tumourigenesis by hedgehog inhibition).

9. Immune cell infiltrate focuses in macrophages and neutrophils, however most other data point to CD8 and MDSC being important. What was the role of these immune cells. Macrophages and neutrophils infiltrate as analysed by authors has shortcomings in terms of false positive staining of a number of other cell types in tissues.

We also stained for CD8 positive T-cells and found that infiltration was also induced in mice treated with LOX blocking antibody and LOX/gemcitabine cotreatment. Our antibodies for both neutrophils and macrophages have previously be validated using approaches to inhibit these populations (e.g. CXCR2 inhibition or antibody depletion for neutrophils and CSF1R inhibition or colodronate for macrophages so we believe our staining protocols are specific (Jamieson *et al* 2012 JCI, Boulter *et al* 2015 JCI).

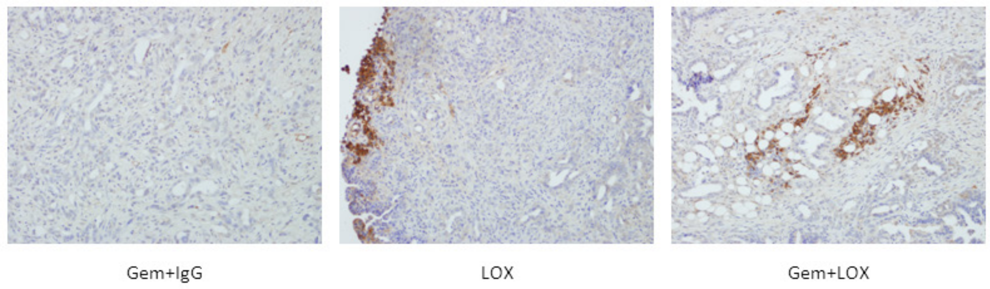

10. Why was Tenascin C evaluated when previous data were on Collagen 1 and Fn.

Along with LOX, TNC was one of a panel of ECM components that were hits in the KPC functional screen. Previous reports have suggested that TNC is overexpressed in pancreatic cancer and have

implicated it in metastasis in breast cancer. As this evidence suggested a possible correlation with LOX, we examined the effects of LOX activity and expression on TNC levels.

11. The absence of metastasis in Lox Ab is not fully evaluated or explained. Why did these mice succumb?

The mice became sick because of primary tumour burden. This is consistent with previous work we have published showing that anti-metastatic effects in the KPC model do not necessarily correlate with increased survival (Morton *et al.* 2010). The effects on the primary tumour when anti-LOX therapy was combined with gemcitabine led to increased survival. We have clarified this in the text.

2nd Editorial Decision 28 April 2015

Thank you for the submission of your re-revised manuscript to EMBO Molecular Medicine. We have now received the enclosed reports from the two Reviewers that were asked to re-assess it. As you will see, while Reviewer 1 is now globally supportive with a few remaining points, Reviewer 2 remains quite critical.

Briefly, Reviewer 2 lists a number of concerns and expresses numerous doubts on the conclusiveness of your findings. Although I have to admit that some of these concerns were not raised in his/her initial evaluation, they do in part originate from the new data presented.

After in depth discussion with my colleagues, we have decided to invite a final revision, although we will not be asking you to provide further experimentation at this point, unless you should have additional data that would strengthen your claims.

Specifically, I would encourage you to 1) send me a point-by-point rebuttal on the points raised by the Reviewers; 2) provide in text and figure clarification as to avoid overreaching or unsupported conclusions on the points mentioned, to clarify experimental/methodological aspects where necessary and to appropriately acknowledge previous work where due and additional supporting data if available; 3) address statistical issues (e.g. Reviewer 2's point 11).

I also suggest that you carefully review the manuscript for other inconsistencies including for instance that the BAPN LOX inhibition (and other) method is still described in this current version even though the data were removed.

I am willing to make an Editorial decision on your final, revised version, provided the issues raised are fully dealt with as mentioned above.

Please also consider the following final Editorial amendments/requests to be included in your revision:

1) Every published paper now includes a 'Synopsis' to further enhance discoverability. Synopses are displayed on the journal webpage and are freely accessible to all readers. They include a short standfirst as well as 2-5 one-sentence bullet points that summarise the paper. Please provide the synopsis including the short list of bullet points that summarise the key NEW findings. The bullet points should be designed to be complementary to the abstract - i.e. not repeat the same text. We encourage inclusion of key acronyms and quantitative information. Please use the passive voice. Please attach this information in a separate file or send them by email, we will incorporate it accordingly. You are also welcome to suggest a striking image or visual abstract to illustrate your article. If you do please provide a jpeg file 550 px-wide x 400-px high.

2) We are now encouraging the publication of source data, particularly for electrophoretic gels and blots, with the aim of making primary data more accessible and transparent to the reader. Would you be willing to provide a PDF file per figure that contains the original, uncropped and unprocessed scans of all or at least the key gels used in the manuscript including the supplementary figures? The PDF files should be labeled with the appropriate figure/panel number, and should have molecular

weight markers; further annotation may be useful but is not essential. The PDF files will be published online with the article as supplementary "Source Data" files. If you have any questions regarding this just contact me.

I look forward to seeing a revised form of your manuscript as soon as possible.

***** Reviewer's comments *****

Referee #1 (Comments on Novelty/Model System):

This is a well done study defining a new pathway controlling PDAC stroma, a cellular compartment with antithetical roles at different stages of this disease. The study also provides strong preclinical evidence supporting the suitability of LOX as a therapeutic target in patients with resectable cases of PDAC.

Referee #1 (Remarks):

The manuscript has sufficiently improved since the initial submission. However, there are few minor details that should be considered to strengthen the conclusions of the study. The control of antibody specificity should be included in the manuscript, the blot of p-SRC should include total SRC expression, in the same figure panel the LOX blot (mature) should be improved. Finally, the discussion on the timing of the in vivo experiments as well as its impact on tumor dynamics should be expanded. This particularly important as multiple trials targeting the stroma are on the way in PDAC and other malignancies.

Referee #2 (Remarks):

Miller et al have improved the manuscript. This allows a more thorough evaluation of their findings and interpretations which was not feasible beforehand. There are several shortcomings which do not justify their abstract or conclusions.
1. APGI analysis. This is informative. ST2 is useful. However, the risk of confounder remains and needs to be evaluated in their in vitro and in vivo models.
a. Did the authors find a correlation between grade or tumor size on LOX expression or Collagen score in GEMM (fig 1F, ST2)
b. How do authors explain tumor location as an independent prognostic marker
c. How does lymph node metastasis and vascular invasion in GEMM correlate with LOX expression or Collagen score
2. What about hypoxia, and collagen score as independent prognostic variables in the model?
3. Numbers at risk are not presented in Fig 1A, 1B, 1D, 1E
4. Risk score for mouse transcriptome not explained in methods or results (Figure 2A).
5. Figures S2C: Dasatinib does not explain LOX. It may explain Scr phosphorylation. The interpretation is therefore unfounded: 'Given our previous data showing a requirement for SRC in KPC cell invasion (Morton et al, 2010b), this suggests that the pro-migratory effects of LOX are mediated at least in part by SRC activation.'
6. shLOX effects on LOX expression are not presented. siRNA effects are partly presented in SF2B: need to show full WB membranes of all the bands. ABT112 is meant to show a single band at ~45KD. In 2D, authors show 31 kD and 55kD. No molecular weights are depicted in SF2B.
7. Slowing of siLOX cells. This needs to be confirmed by cell cycle analysis. Explanation, with evidence, should be provided why cell-cycle is affected.
8. In the 920 siRNA screen, after siRNA with LOX, Fn and TnC (ST3), there was a reduction in cell viability. However in contrast, upon siRNA LOX, Fn expression was increased (SF3C). How can this be reconciled. Surprisingly siFn affected cell viability (SF3: unnumbered or SF3C bottom).
9. The specificity of LOX function-blocking antibody is not provided in Cox et al 2013 as cited.
10. Data on mice used in Figure 3D onwards such as number, age are not provided.
11. If the data on mice is less than n=15 per cohort, then data in Figu4B-D should be provided at individual mouse level rather than box-plots.

12. Despite authors' assertion of specificity of f4/80 and MPO in Figure 4A, there is a lot of non-specific staining.
13. The introduction still remains over-generous and not-pertinent to main them of the paper. For example there is no background on Baker and Cox papers which are leading articles in the field. Most of what is shown in this paper has been shown by these authors in other models.

Finally a question of interpretation as shown in abstract.
a. There is no effect of LOX blockade in late-stage tumours.
b. There is effect of LOX blockade in early stage tumors: in authors' words representing those with resectable disease.
c. Gemcitabine or other neo-adjuvant treatment is not given (as a routine outside clinical trials) to patients with operable disease.
Hence abstract has to be substantially modified to reflect:
1. Limitations of this approach in context of human disease.
2. Specify stromal alterations and type of immune cell infiltrate
3. No evidence of suppression of metastasis from synergetic effect. This seems to be limited to LOX antibody treatment alone.

2nd Revision - authors' response 12 May 2015

Referee #1 (Comments on Novelty/Model System):

Referee #1 (Remarks):

The manuscript has sufficiently improved since the initial submission. However, there are few minor details that should be considered to strengthen the conclusions of the study. The control of antibody specificity should be included in the manuscript, the blot of p-SRC should include total SRC expression, in the same figure panel the LOX blot (mature) should be improved. Finally, the discussion on the timing of the in vivo experiments as well as its impact on tumor dynamics should be expanded. This particularly important as multiple trials targeting the stroma are on the way in PDAC and other malignancies.

With regard to antibody specificity, this has been addressed in:

Baker et al JNCI 2011: antibody recapitulates effects of LOX shRNA in colon cancer cells in terms of effects on proliferation, invasion, tumour growth and metastasis.

Baker et al Cancer Research 2013: titration of LOX antibody against recombinant protein in activity assay.

Cox et al Cancer Research 2013: Binding assays show antibody binds recombinant LOX protein and not recombinant LOXL2 protein.

Li et al 2009: LOX antibody specificity confirmed by peptide blocking studies.

We have now included these references.

The lower panel of Figure S2B showed the blot for total Src, but we have reordered the panels to make it clearer. We have also substituted the blot of the lower LOX band for a better version.

We already expanded the discussion on the timing of *in vivo* experiments with mention of other stromal-targeting trials, in the main text. We have now added additional discussion in the discussion section.

Referee #2 (Remarks):

Miller et al have improved the manuscript. This allows a more thorough evaluation of their findings and interpretations which was not feasible beforehand. There are several shortcomings which do not justify their abstract or conclusions.

1. APGI analysis. This is informative. ST2 is useful. However, the risk of confounder remains and needs to be evaluated in their in vitro and in vivo models.

There is always a risk of confounders but experiments were conducted to minimise this risk. Even patients from randomised controlled trial cohorts have a selection bias, based on inclusion and exclusion criterion. The APGI cohort is a community-acquired cohort, however, consecutive patients treated in participation hospitals were recruited and there was no refusal of consent.

a. Did the authors find a correlation between grade or tumor size on LOX expression or Collagen score in GEMM (fig 1F, ST2)

We were quite confused by this comment, since there is no GEMM data in fig 1F or S2. We have tried to address what we think the questions might be:

Related to figure 1F: We did not find a correlation between LOX expression and tumour grade/stage, because LOX has already crosslinked the collagen present in these tumours.

Related to figure S2: The experiments in figure S2 are not conducted in GEMM, but in the xenograft model. However, in the GEMM model many initiating lesions give rise to several tumours in the pancreas that converge upon each other and grade is never assessed. Also, since all mice are sacrificed because of tumour burden, and not at time-point, we did not compare tumour size.

b. How do authors explain tumor location as an independent prognostic marker

It is a well-known phenomenon that tumours of the body/tail of the pancreas tend to have poorer prognosis than tumours of the head, even adjusting for stage, grade and other pathological variables. This has been described in several studies; however, the reasons for this are unknown. Some hypothesise that these tumours may be more likely to harbour occult metastatic disease or metastasise earlier, or that they may represent more aggressive disease.

c. How does lymph node metastasis and vascular invasion in GEMM correlate with LOX expression or Collagen score

Vascular invasion has never been shown for this model, and we do not observe frank vascular invasion. We have not statistically correlated lymph node metastasis with LOX expression or collagen score since LN metastases are very rare. We do however describe (and show in Figure 2A) that multiple members of the Lox family are overexpressed in metastatic disease compared with non-metastatic disease.

2. What about hypoxia, and collagen score as independent prognostic variables in the model?

Mulltivariate analysis is suited to human cohorts but is never performed in mice. The number of mice that would be required for multivariate analysis of would be far larger than the sample sizes used in this, or any other study, and due to adherence to the principles of the 3Rs and Home Office regulations we would not be permitted to generate cohorts of that size.

3. Numbers at risk are not presented in Fig 1A, 1B, 1D, 1E

We have now presented these data.

4. Risk score for mouse transcriptome not explained in methods or results (Figure 2A).

Details were given in the figure legend.

They were explained in the legend of figure 2a. We have additionally clarified that the loading values used came from the PC-1 signature analysis.

5. Figures S2C: Dasatinib does not explain LOX. It may explain Scr phosphorylation. The interpretation is therefore unfounded: 'Given our previous data showing a requirement for SRC in KPC cell invasion (Morton et al, 2010b), this suggests that the pro-migratory effects of LOX are mediated at least in part by SRC activation.'

We don't believe that there is anything wrong with this statement, given that we show here that reducing LOX expression results in loss of Src phosphorylation and we have previously shown a requirement of phospho-Src for KPC cell invasion. We are, however, happy to amend this statement to read "Given our previous data showing a requirement for SRC in KPC cell invasion (Morton et al, 2010b), this suggests that the pro-migratory effects of LOX could be mediated at least in part by SRC activation."

6. shLOX effects on LOX expression are not presented. siRNA effects are partly presented in SF2B: need to show full WB membranes of all the bands. ABT112 is meant to show a single band at ~45KD. In 2D, authors show 31 kD and 55kD. No molecular weights are depicted in SF2B.

Westerns for the effect of shLOX are now included in Figure S2F and molecular weights added to the figures. The multiple bands detected for LOX represent the prepro and mature form as shown in numerous publications for ABT112. In addition we confirmed their identity by siRNA-mediated knockdown.

7. Slowing of siLOX cells. This needs to be confirmed by cell cycle analysis. Explanation, with evidence, should be provided why cell-cycle is affected.

We have not performed cell-cycle analysis and did not report that cell cycle was affected. We simply reported a reduction in the number of viable cells without an increase in apoptotic cells, and suggested that this was "presumably due to a slowing of cell growth".

We have now removed this suggestion from the text.

8. In the 920 siRNA screen, after siRNA with LOX, Fn and TnC (ST3), there was a reduction in cell viability. However in contrast, upon siRNA LOX, Fn expression was increased (SF3C). How can this be reconciled. Surprisingly siFn affected cell viability (SF3: unnumbered or SF3C bottom).

Again, we were slightly confused by this comment. There are hundreds of examples in biology of compensatory upregulation of one protein following knockdown of another. We believe that the upregulation of Fn in response to LOX inhibition is a compensatory mechanism. However, it clearly doesn't automatically follow that this upregulation of Fn is sufficient to overcome the loss of viability caused by siLOX.

9. The specificity of LOX function-blocking antibody is not provided in Cox et al 2013 as cited.

We have added further citations to address this as outlined in response to reviewer 1.

10. Data on mice used in Figure 3D onwards such as number, age are not provided.

The inclusion of Kaplan-Meier analysis, by definition, provides data on age of mice at end-point, while for all the figures involving mice, numbers are already included. In the legend for Figure 3D we clearly include number of mice (in brackets, as n=X), and clearly state for Figure 3F that at least 5 mice were scored per group. The same is included in the legend for figure 4, and now that individual data point plots are included the numbers are evident in the figure as well.

11. If the data on mice is less than n=15 per cohort, then data in Figu4B-D should be provided at individual mouse level rather than box-plots.

We have now replaced the boxplots with graphs showing individual data points.

12. Despite authors' assertion of specificity of f4/80 and MPO in Figure 4A, there is a lot of non-specific staining.

We have included (as a reviewer figure), F480 and MPO staining in pancreas following neutrophil or macrophage depletion, to demonstrate the specificity of these antibodies. The staining that the reviewer refers to as non-specific is not non-specific and is characteristic of immune cell infiltration in the PDAC stroma.

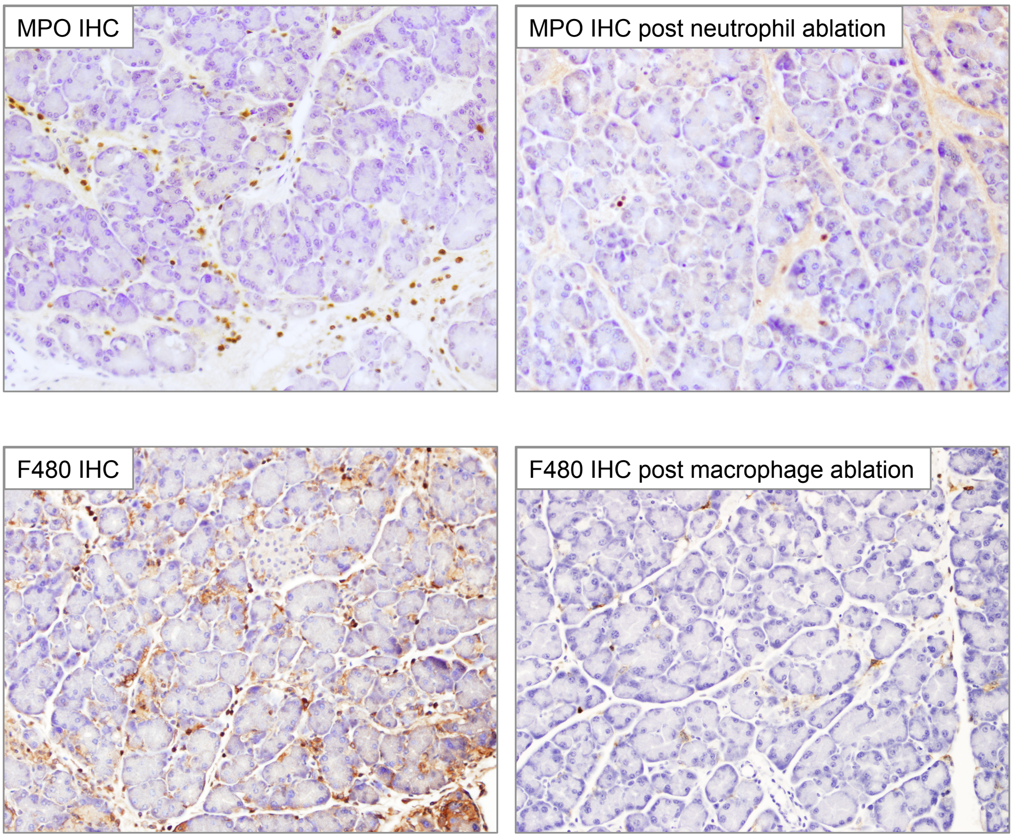

13. The introduction still remains over-generous and not-pertinent to main them of the paper. For example there is no background on Baker and Cox papers which are leading articles in the field. Most of what is shown in this paper has been shown by these authors in other models.

In an effort not to 'self-cite' too much as requested by the reviewer in the first review we did not cited all the Baker and Cox papers as they are from the lab of the joint corresponding author, J Erler. We have now cited additional papers.

Finally a question of interpretation as shown in abstract.

a. There is no effect of LOX blockade in late-stage tumours.

b. There is effect of LOX blockade in early stage tumors: in authors' words representing those with resectable disease.

c. Gemcitabine or other neo-adjuvant treatment is not given (as a routine outside clinical trials) to patients with operable disease.

Hence abstract has to be substantially modified to reflect:

1. Limitations of this approach in context of human disease.

We have included the fact that mice with early stage tumours respond, and proposed that LOX inhibition *could* improve outcome specifically in surgically resectable disease.

2. Specify stromal alterations and type of immune cell infiltrate

We have now added these details to the abstract.

3. No evidence of suppression of metastasis from synergetic effect. This seems to be limited to LOX antibody treatment alone.

We clearly state in the abstract that LOX antibody suppresses metastasis and then go on to describe the additional synergistic effect of LOX inhibition and gemcitabine on survival.

© EMBO
